# Identification of heterogeneous subtypes of human dendritic cells from the lymph nodes of patients with gastric cancer

**DOI:** 10.3389/fimmu.2026.1912586

**Published:** 2026-10-02

**Authors:** Song-Hee Han, In-Jeong Lee, Hue Vy An Tran, Mi Ha Joo, Kwan Woo Kim, JungMo Kim, Seung Ho Kim, Hyun Goo Woo, Min Chan Kim, Jong-Young Kwak

**Affiliations:** 1 Department of Pathology, College of Medicine, Dong-A University, Busan, Republic of Korea; 2 3D Immune System Imaging Core Center, Ajou University, Suwon, Republic of Korea; 3 Department of Pharmacology, School of Medicine, Ajou University, Suwon, Republic of Korea; 4 Department of Surgery, College of Medicine, Dong-A University, Busan, Republic of Korea; 5 Ajou Translational Omics Center (ATOC), Research Institute for Innovative Medicine, Ajou University Medical Center, Suwon, Republic of Korea; 6 Department of Physiology, School of Medicine, Ajou University, Suwon, Republic of Korea

**Keywords:** dendritic cell, epitope-sequencing, gastric cancer, lymph node, transcriptomes

## Abstract

**Objective:**

The subtypes of heterogeneous conventional dendritic cells (cDCs) and plasmacytoid dendritic cells (pDCs) in lymph nodes (LN) cannot be distinguished because they share similar surface markers. This study aimed to identify cDCs, pDCs, and other cell types in DC-enriched LNs from patients with gastric cancer (GC) by analyzing surface markers and gene expression.

**Methods:**

Expression of surface markers, including CD141, CD1c, and CD123, in DC-enriched cells was analyzed by conventional flow cytometry and AbSeq using antibody-derived tags. Transcriptome and epitope analyses were used to characterize DC subtypes. The frequencies of heterogeneous DC subtypes were compared across clinical stages and molecular subtypes in patients with GC.

**Results:**

DC-enriched cells showed CD1c^+^CD141^+^ and CD141^+^CD123^+^ populations. A clustering approach based on antibody-derived tags identified 17 clusters with single- and double-positive DC surface markers. Transcriptome and epitope analyses identified 26 clusters with distinct surface markers and gene expression profiles. DC lineages included subtypes distinct from the typical cDC1, cDC2, and pDC types. A proportion of CD1c-expressing cDC2 lineage cells expressed surface features characteristic of cDC1 and pDC, and CD1c expression was observed in cell types other than DC lineages. Differences in DC cluster frequencies across DC lineages were not detected as significant across clinical stages and molecular subtypes of GC. The mRNA expression pattern in the CD141^+^ cDC1 lineage distinguished it from the CD1c^+^ cDC2 lineage and resolved different clusters with transitional states within both lineages. Transcripts related to DC maturation and migration were detected in a CD137-DC subtype, which highly expressed HLA-DR and CD137.

**Conclusion:**

Cluster analysis of DC-enriched cells in LNs from our integrated dataset revealed subtypes of the cDC1, cDC2, and pDC lineages. We identified distinctive DC subtypes in the transitional state bridging differentiation between cDC1 and cDC2. CD137-DC may be an independent DC subset with mature and migratory features.

## 1 Introduction

Dendritic cells (DCs) comprise multiple subtypes, including conventional DCs (cDCs) and plasmacytoid DCs (pDCs), which are distinguished by their surface marker expression and transcriptional profiles (1). Human cDCs are the traditional antigen-presenting DCs and can be further subdivided by CD141 and CD1c expression into cDC1 and cDC2, respectively (2, 3). In humans, pDCs are identified by CD123 expression (1). Human DC types across lymphoid organs are primarily characterized by cell-type-specific transcriptional programs rather than by the tissue microenvironment (4).

Villani et al. identified other previously undescribed DC subsets, including DC3, DC4 (CD141^−^CD1c^−^), and a rare DC5 (AXL^+^SIGLEC6^+^ AS-DC) subset (5). Transitional DC (tDC), also known as AS-DCs, is a hybrid subset that bridges pDCs and cDC2s and has distinct developmental features (5–7). tDCs express genes associated with pDCs and cDC2s and exhibit surface marker expression, with some cells expressing high levels of pDC markers and others expressing high levels of cDC markers (6, 7). DC3s, which share phenotypic markers with cDC2s and monocytes, have been identified as an inflammatory DC population (5). This population is functionally different from both DC2s and monocytes (8). In addition, a distinctive DC subtype has been identified. The CD137^+^ (4-1BB^+^) DC subtype was found in the mouse spleen, and CD137 engagement in DCs leads to cytokine secretion and improved ability to stimulate T cell responses (9, 10).

DCs in the tumor microenvironment (TME) are essential components that promote antitumor T-cell responses (11). cDC1s specialize in antitumor immune responses by cross-presenting tumor-associated antigens to CD8^+^ T lymphocytes (12). cDC1 cells in the tumor-draining lymph node (LN) maintain a reservoir of CD8^+^ T cells and support antitumor immunity (13). Moreover, cDC1 transcriptional profiles and frequency are associated with improved survival and enhanced treatment responsiveness in cancer patients (14). The role of cDC2 cells in cancer immunology is less well established than that of cDC1 cells, likely due to their heterogeneity and the lack of specific cell markers for identification (11). Higher pDC frequencies at the tumor site are associated with poor prognosis (4). DC3s (CD1c^+^CD5^-^CD163^+^CD14^±^) display functional plasticity between anti-tumor defense and cancer-mediated immunosuppression depending on the solid tumor type (15). Recent work revealed a specialized tolerogenic role for tDC2s by inducing the generation of GATA3-expressing effector T_reg_ cells in inflammation and cancer (16).

Gastric cancer (GC) is the fourth leading cause of cancer-related death worldwide (9). GC is a malignant tumor characterized by diverse immune infiltrates, and immune cells in the TME may influence the efficacy of GC immunotherapy (17). Several cancers have shown that cDC1 tissue density is associated with therapeutic response and overall patient survival (14). GC cohorts showed that a high cDC1-related gene signature was associated with shorter overall and disease-free survival in the Cancer Genome Atlas (TCGA) and Gene Expression Omnibus (GEO) datasets (18). However, a signature derived from bulk tumor tissue does not establish which cells carry the transcripts, and the analysis was confined to the primary tumor, not to cDC1s that prime CD8^+^ T cells in the draining LN. Resolving what these markers label and which compartment they mark requires characterization at the DC subtype level.

Characterization of DC subtypes in patients with cancer remains unexplored because of their heterogeneity and the lack of clear markers to identify each subtype. Single-cell RNA sequencing (scRNA-Seq) has aided cell-type identification, but identifying multiple DC subtypes in scRNA-Seq studies is primarily based on transcriptional state (5). Compared with mapping tumor-infiltrating immune cells using scRNA-Seq, DC heterogeneity in the LNs of cancer patients remains unexplored. The method, cellular indexing of transcriptomes and epitopes by sequencing (CITE-Seq), combines highly multiplexed antibody (Ab)-based detection of surface markers with single-cell transcriptome profiling (19). To more fully characterize DC phenotypes in lineage-negative and HLA-DR-positive (Lin^-^HLA-DR^+^) DC-enriched cells from LNs of patients with GC, we identified several DC subtypes using antibody-derived tags (ADTs) and RNA expression by scRNA-Seq. This approach provides insights into the heterogeneity of human DC subtypes in LNs that share many surface and genetic markers.

## 2 Materials and methods

### 2.1 Patients with GC

Clinical data and LN specimens (n = 80) were obtained from 12 patients with GC who underwent gastrectomy at Dong-A University Hospital (Busan, Korea) in 2022. Only LNs without metastatic involvement were included in this study, as confirmed by intraoperative frozen section examination to exclude tumor cell contamination. The detailed clinicopathological characteristics of GC and LNs are summarized in **Supplementary Table S1**. Clinicopathological information, including age, sex, and tumor stage, was retrieved from surgical pathology reports. Tumor staging was performed according to the eighth edition of the American Joint Committee on Cancer Staging Manual. Control LNs were obtained from patients without malignancy undergoing cholecystectomy for benign gallbladder disease at the same institution between December 2022 and April 2023. We analyzed three LNs by flow cytometry; we processed one for CITE-seq and used a separate LN for immunohistochemistry. All patients provided written informed consent for tissue collection. The Institutional Review Board of Dong-A University Hospital approved the study protocol (DAUHIRB-22-007; December 6, 2022), and the study was conducted in accordance with the Declaration of Helsinki.

### 2.2 Isolation of total LN and DC-enriched cells

Regional LNs were taken from the perigastric stations along the lesser and greater curvatures and from the extraperigastric stations No. 8a (anterosuperior group along the common hepatic artery) and No. 9 (celiac artery), classified according to the Japanese Gastric Cancer Association based on their location around the stomach in GC patients (**Supplementary Table S2**) (20). In the pathological staging, metastatic cancer cell-negative LNs were used. Fresh LN specimens were mechanically minced into small fragments and enzymatically dissociated in RPMI-1640 medium containing collagenase type IV (0.5 mg/mL; Thermo Fisher Scientific, Waltham, MA, USA) and DNase I (20 μg/mL; Sigma-Aldrich, St. Louis, MO, USA) for 30 min at 37 °C. Enzymatic digestion can affect transcriptional changes in certain genes, such as genes encoding heat-shock proteins, but we used it to prevent extremely low DC yields and poor subtype representation after insufficient tissue digestion by mechanical dissociation (21). Cell suspensions were filtered through a 40 μm cell strainer and subjected to red blood cell lysis. The average total LN cell yield was 3.7 × 10^7^ cells. DC-enriched cells were prepared using a human Pan-DC Enrichment Kit (Miltenyi Biotec, Bergisch-Gladbach, Germany) according to the manufacturer’s instructions, yielding 5 × 10^5^ Lin^-^HLA-DR^+^ cells per LN. The total LN cell fraction was used for the flow cytometric analysis, and the DC-enriched fraction was reserved for AbSeq and scRNA-Seq analysis.

### 2.3 Flow cytometry

For phenotypic analysis, the cells were stained with fluorochrome-conjugated Abs against lineage and DC-associated markers, as listed in **Supplementary Table S3**. Flow cytometric acquisition was performed using the spectral flow cytometer Aurora 3 (Cytek Biosciences, Fremont, CA, USA), and data were analyzed using FlowJo™ software (version 10.9.0). Gates were defined and visually verified manually for each sample before any automated analysis. Viable leukocytes were gated as CD45^+^ cells after exclusion of debris, doublets, and dead cells; B and T cells were gated out using Abs against CD19 and CD3; CD14^+^ and CD11b^+^ cells were gated out from CD3^-^CD19^-^HLA-DR^+^ cells; and CD3^-^CD19^-^CD14^-^CD11b^-^ (Lin^-^) and HLA-DR^+^ cells were subsequently analyzed for the expression of CD141, CD1c, CD123, and other DC-related markers. We inspected and adjusted gate positions per sample rather than applying a single template gate across samples. After this manual verification, we selected 3,000 cells per sample using the DownSampleV3 plugin in FlowJo, concatenated them, and analyzed them by t-distributed stochastic neighbor embedding (t-SNE) using default settings.

### 2.4 scRNA and protein profiling

To assess surface marker overlap across DC subtypes, simultaneous single-cell transcriptomic and proteomic profiling was performed on the BD Rhapsody platform using whole-transcriptome analysis (WTA) and AbSeq. DC-enriched cells were stained with the BD AbSeq Immune Discovery Panel, comprising 30 pre-titrated Abs, along with three additional Abs specific for DC subtypes (**Supplementary Table S4**). Four multiplexed single-cell library/sequencing batches, designated DC-set01 to DC-set04, were generated from 12 LN specimens. Each batch comprised three independently tagged LN cell suspensions. The assignment of each patient ID, anatomical LN location, Sample Tag, clinical stage, and molecular subtype to the four batches is provided in **Supplementary Table S2**. The 12 LN specimens represent seven unique patient IDs because multiple anatomical LN specimens were analyzed from some patients. RNA and AbSeq data were integrated separately across the four runs using reciprocal principal component analysis (rPCA)-based anchor integration before construction of the multimodal WNN graph. In the same 70,465 cells, the normalized 30-nearest-neighbor run-mixing ratio increased from 0.433 to 0.814 for RNA and from 0.468 to 0.826 for AbSeq, while the mean marginal fraction of principal-component variance attributable to run decreased from 0.0469 to 0.0221 for RNA and from 0.0988 to 0.0132 for AbSeq. The corresponding before/after UMAPs are provided in **Supplementary Figure S1**. Twenty-five of the 26 annotated populations were detected in all four runs. AbSeq libraries were sequenced to a target depth of 30,000 reads per cell using an Illumina NovaSeq 6000 platform (Macrogen, Seoul, Republic of Korea). Single-cell libraries were generated using BD Rhapsody WTA, AbSeq, and Sample Tag workflows according to the manufacturer’s instructions. Primary processing was performed using the BD Rhapsody Sequence Analysis Pipeline on the Seven Bridges Genomics Cloud platform. Gene expression was quantified using the BD Rhapsody human WTA reference package, based on GRCh38. Initial visualization and quality assessment were performed using SeqGeq and DataView.

### 2.5 Data processing and quality control

The cells were analyzed using RNA, ADT, and joint-weighted nearest-neighbor (WNN) representations. Quality control was performed separately for each processing/sequencing run before data integration and clustering. The exact run-specific nFeature_RNA and percent.mt thresholds are listed in **Supplementary Table S5**. DC-set01 retained cells with 1,000 < nFeature_RNA < 7,500 and percent.mt < 30%; DC-set02 and DC-set03 retained cells with nFeature_RNA > 500 and percent.mt < 30%, without an upper nFeature_RNA cutoff; and DC-set04 retained cells with 500 < nFeature_RNA < 6,000 and percent.mt < 30%. Thresholds were selected by visual inspection of the pre-integration nFeature_RNA, nCount_RNA, and percent.mt distributions. nCount_RNA was evaluated diagnostically but was not used as an exclusion criterion. Cells classified by Sample Tag demultiplexing as cross-sample multiplets or undetermined were excluded. After run-specific RNA QC and Sample Tag filtering, 71,283 singlets entered WNN analysis; removal of two artifactual clusters containing 818 cells yielded a final dataset of 70,465 cells. Sample Tag demultiplexing was performed using the BD Rhapsody Sequence Analysis Pipeline v2.0. After tag-specific background estimation and noise subtraction, a cell exceeding the calling threshold for one Sample Tag was assigned as a singlet, a cell exceeding thresholds for two or more Sample Tags was classified as a cross-sample multiplet, and a cell exceeding no tag-specific threshold was classified as undetermined. Only cells that passed RNA QC and carried a single Sample Tag assignment were retained. This procedure does not identify same-sample doublets. The run-specific counts and percentages are listed in **Supplementary Table S6**.

### 2.6 Data integration and clustering

Batch correction was performed independently for the RNA and ADT modalities using rPCA-based integration implemented in Seurat. For RNA, each dataset was normalized, 2,000 highly variable genes were identified, and integration was performed across four sequencing runs using rPCA-based anchor detection. For ADT, the counts were normalized using centered log-ratio (CLR) normalization before rPCA-based integration. The resulting batch-corrected RNA and ADT assays were used for the multimodal analysis. Multimodal integration was performed using WNN analysis. The RNA (PCs 1–13) and ADT (PCs 1–8) embeddings were combined to generate a joint WNN-uniform manifold approximation and projection (UMAP) from the WNN graph. Clustering was evaluated across a range of resolution parameters, and transitions between adjacent resolutions were visualized using the clustree R package (22). The clustering trees for all three representations are provided as **Supplementary Figure S2**, and the number of clusters obtained at each resolution is given in **Supplementary Table S7**. Final resolutions were selected by considering cluster persistence, cluster size, and the interpretability of RNA and ADT marker patterns. RNA-only and ADT-only analyses used Louvain clustering (algorithm = 1) at resolutions 0.5 and 0.4, respectively. The primary WNN analysis used the smart local moving SLM algorithm (algorithm = 3) at resolution 0.6.

### 2.7 Protein-level analysis

The AbSeq-derived protein counts were CLR-normalized and integrated across batches using rPCA. DC subtypes and intermediate populations co-expressing multiple DC markers were defined using empirically determined CLR-normalized ADT expression thresholds. Protein expression was visualized using WNN-UMAP embedding, and average protein expression was summarized across annotated cell populations and clinical metadata groups.

### 2.8 Differential gene expression analysis

To characterize transcriptomic features of DC subtypes, cluster-enriched marker genes were identified from the integrated RNA assay using the nonparametric Wilcoxon rank-sum test with an adjusted *p*-value < 0.05, a log2 fold change > 0.25, and a minimum detection fraction of 0.25. Selected transcription factors and toll-like receptor (TLR) genes relevant to DC biology were examined across annotated populations for downstream visualization (23).

### 2.9 Statistical analysis

Flow cytometric data were analyzed at the level of individual lymph nodes (n = 33) and are presented as mean ± SD. Because the four subsets defined by each marker pair are mutually exclusive fractions of the same LN, they were compared by repeated-measures one-way ANOVA, with Greenhouse–Geisser correction when Mauchly’s test indicated a violation of sphericity, followed by paired comparisons with Holm correction. Frequencies of two different clusters were compared between CIN (n = 6) and GS (n = 4) samples using Welch’s two-tailed t-test; the exact Mann–Whitney U test was applied as a non-parametric confirmation. A p-value of less than 0.05 was considered statistically significant. Differences in frequency among the three cDC1 clusters were assessed using the Friedman test with *post hoc* Wilcoxon signed-rank tests and Holm correction for multiple comparisons (n = 12). Comparisons of frequency between the AGC and EGC groups were performed using the two-tailed Mann–Whitney U test, as the data did not satisfy the assumption of normality (Shapiro–Wilk test, p < 0.01). Exact p-values were computed given the small sample size. Data are presented as mean ± SD. A p-value < 0.05 was considered statistically significant. All analyses were performed using GraphPad Prism version 10.4.0 (GraphPad Software, Boston, MA, USA). Analyses of ADT and transcriptome data were performed in R (v4.3.1) using Seurat (v4.3.0) and ggplot2 (v3.4.4).

### 2.10 Histologic examination and quantification of LNs

Tumor-draining LNs from GC patients and one control LN were fixed in 10% neutral buffered formalin and embedded in paraffin. Immunohistochemical staining was carried out on whole-slide sections of the LNs, selected based on histopathological evaluation and confirmed to be free of metastatic involvement. Immunostaining was performed using a BenchMark XT automated immunostainer with an UltraView Universal DAB Detection Kit (Ventana Medical Systems Inc., Tucson, AZ, USA). Following deparaffinization, rehydration, and antigen retrieval, sections were incubated with the following primary antibodies: CD141 (1:100, Abcam, Cambridge, UK), CD1c (1:100, Novus Biologicals), CLEC9A (1:80, Abcam), CLEC10A (1:80, OriGene, Rockville, MD, USA), S100B (1:100, Abcam), and CD137 (1:150, Abcam).

Slides were scanned at ×40 and analyzed in QuPath v0.6.0. Annotation and scoring were done by one board-certified pathologist, blinded to stage and molecular subtype. Two compartments were scored: paracortex, taken as the T-cell zone including the interfollicular areas, and lymphoid follicle, outlined in whole with its mantle zone. Medulla was not scored, as it was absent from the section plane in several cases. Two regions were annotated per compartment on each slide, yielding eight fields per compartment for each antibody. Region areas were recorded and varied with the size of the structure sampled. Capsule, subcapsular sinus, and artifactual areas were avoided, and matching compartments were taken on the serial sections of each antibody. Color deconvolution vectors were estimated per antibody and applied to all four sections of that antibody. The hematoxylin vector was kept constant across antibodies, since sections with little unstained lymphoid tissue gave unstable estimates. Cells were scored with the positive cell detection command (detection image, optical density sum; requested pixel size, 0.5 µm; nuclear threshold, 0.1; cell expansion, 5 µm), using a single intensity threshold on the mean DAB optical density of the cell. Thresholds were set per antibody against unequivocally stained cells and kept unchanged across the four sections. Densities are given as positive cells per mm². Paracortex and follicle were compared by Mann-Whitney U test with Benjamini-Hochberg correction across antibodies (R v4.3.1); adjusted p < 0.05 was considered significant. Densities rather than percentages are reported, as counterstain intensity varied between sections and negative nuclei were not reliably detected on all slides.

## 3 Results

### 3.1 Clinicopathological characteristics of the study cohort and LN sampling

This study included 12 patients with GC. Tumors were most frequently located in the lower third of the stomach (50.0%), followed by the middle third (33.3%) and the upper third (16.7%). Histologically, tubular adenocarcinoma was the predominant subtype (58.3%). According to the Lauren classification, intestinal-type tumors were most common (58.3%), followed by diffuse (16.7%) and mixed or indeterminate types (25.0%). By tumor depth, four patients (33.3%) had early GC (EGC), and eight (66.7%) had advanced GC (AGC). Most tumors were microsatellite stable (MSS, 75.0%), with a minority exhibiting high microsatellite instability (MSI-H, 25.0%).

### 3.2 Fraction of DC-enriched cells by flow cytometry

Lin^-^HLA-DR^+^ cells were selected from the CD3^−^CD19^−^CD14^−^ cells and further gated to identify the CD123^+^ and CD141^+^ populations (**Figure 1A**). The CD1c^-^CD123^+^ G1 fraction contained pDCs. pDCs were 14.7 ± 7.8% of the DC-enriched cells and 1.13 ± 0.10% of the total LN cells (**Supplementary Table S8**). The percentage of DC-enriched cells in LN from patients was lower than in control LN (0.79 ± 0.35 vs. 2.65 ± 0.25, *p* < 0.01) (**Supplementary Table S9**). However, the percentage of each fraction did not differ significantly between the control and patient groups. CD1c^+^CD141^+^ double-positive cells were more than 50% of the CD123^-^CD141^+^ cells (G2 fraction), and CD1c^-^CD141^-^ double-negative cells were more than 75% of the CD123^-^CD141^-^ cells (G3 fraction). Thus, CD123-negative cells (G2 and G3 fractions) were subdivided into four subsets by expression of CD141 and CD1c as follows: CD141^+^CD1c^+^ (F1), CD141^+^CD1c^-^(F2), CD141^-^CD1c^+^(F3) and CD141^-^CD1c^-^ cells (F4). Among G2 and G3 fractions, CD141^+^CD1c^-^(F2) and CD141^-^CD1c^+^(F3) were 25.2 ± 8.9% and 10.1 ± 7.5%, respectively, because double-positive and double-negative cells were commonly detected (**Figure 1B**). CD141^+^ cells co-expressed CD1c and CD123, and CD141^high^ cells lacking double-positive markers accounted for < 5% of cells. Boolean combination gating analysis of 8 populations also confirmed the frequencies of single-positive, double-positive, and double-negative cells among the Lin^-^HLA-DR^+^CD14^-^CD11b^-^ population (**Supplementary Figure S3**). Human cDC1 cells contain both XCR1^+^ and XCR1^-^ cells (24). XCR1^+^ cells were mainly detected in F2, and a small proportion of F4 cells also contained XCR1^+^ cells (**Figure 1C**). The expression of programmed cell death ligand 1 (PD-L1) was analyzed in all fractions. The XCR1-expressing F2 fraction was the main source of PD-L1-expressing DCs. t-SNE analysis of multicolor flow cytometry data revealed several clusters expressing DC surface markers; however, CD1c^+^ and CD141^+^ cells could not be clearly distinguished (**Figure 1D**). Double-positive clusters, including CD1c^+^CD141^+^ and CD141^+^CD123^+^ clusters, were visualized at high frequency, and CD1c expression was not detected in the CD141^+^XCR1^+^ cluster in the heatmap or histograms. Double-positive cells may be a hybrid transitional subset similar to transitional DCs that explicitly bridges pDCs and cDCs (7, 25). The CD1c^+^CD141^+^ cluster may represent a transitional cDC subtype that bridges the differentiation stage between cDC1 and cDC2 in LN.

**Figure 1:**
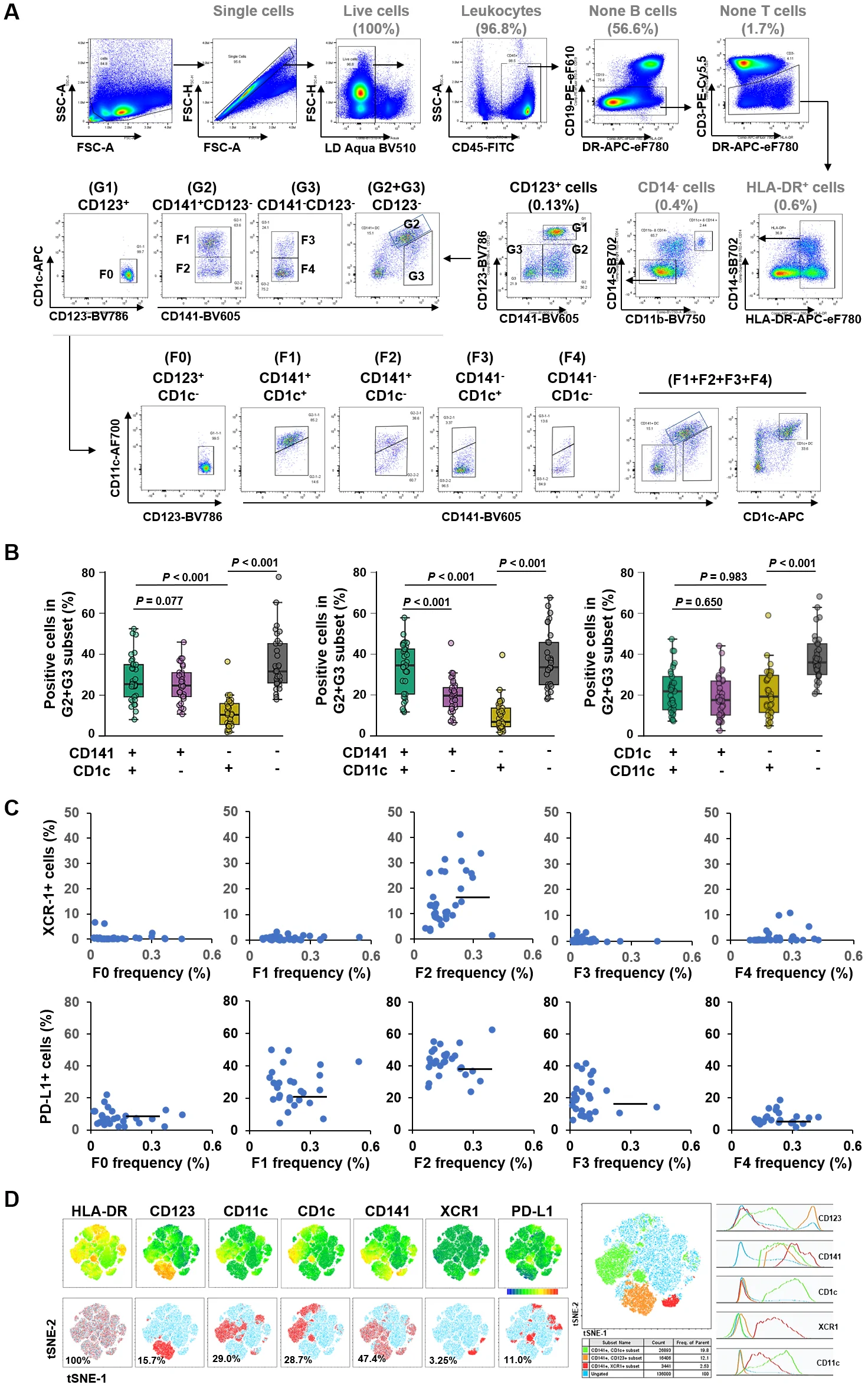
Manual gating for the identification of DC subtypes from LN. **(A)** Representative gating strategy from total LN cells (n = 33) to define CD123^+^, CD141^+^, and CD1c^+^ cells in the Lin^-^HLA-DR^+^ population. Grey percentages above each plot indicate the frequency of the gated population within its parent gate. **(B)** Frequencies of cells positive for the indicated CD141/CD1c, CD141/CD11c, or CD1c/CD11c marker combinations within the combined G2+G3 compartment. Points represent individual LNs (n = 33), and boxes summarize the distributions. p-values above the brackets were calculated using one-way ANOVA. **(C)** Frequency of XCR1^+^ (upper) and PD-L1^+^ (lower) cells in the CD141^+^CD1c^-^ fraction (n = 33). Horizontal bars indicate the mean. **(D)** t-SNE analysis of multicolor flow cytometry data. In the upper row of the left panel, a sequential color gradient from green to red maps expression levels of specific surface markers; positive cells are shown in red in the lower row. In the middle panel, each subset was manually defined on the same t-SNE map. The right panel shows histograms of surface marker expression levels for these subsets, colored as in the middle panel.

### 3.3 Expression of surface markers on DCs detected by AbSeq

AbSeq analysis was performed using the BD^®^ AbSeq Immune Discovery Panel, supplemented with barcoded Abs against DC-associated surface markers. Cells were clustered based on ADT expression profiles using an unsupervised, graph-based clustering algorithm (SLM). The resulting clusters were visualized on the ADT data using UMAP (**Figure 2A**; **Supplementary Figures S4A and S4B**). UMAP showed that CD1c^+^, CD123^+^, and CD141^+^ cells formed distinct clusters (**Figure 2B**). Unsupervised Leiden clustering of the integrated ADT data identified 17 clusters (C0–C16). These clusters exhibited distinct surface marker expression profiles: C2, C8, and C10 expressed CD141, C1, C2, C3, and C8 expressed CD1c, and C0, C11, and C15 expressed CD123 (**Figure 2C**). C11 and C12 expressed CD141, CD1c, and CD123. Dot plot analysis revealed differential surface marker expression across all clusters, with varying percentages of cells in each cluster (**Figure 2D**). The proportion of CD123-expressing cells in C_0_ exceeded 30%. CD1c^+^CD11c^+^CD141^+^ C_2_ cells accounted for 13% of the total. A proportion of C8 cells expressed CD141 and Tim3, accounting for 5% of the total DC-enriched cells. In addition to the established DC subtypes, C1 showed high HLA-DR and CD137 expression, suggesting that C1 belongs to the CD137^+^ DC subtype (13%). UMAP plots for each cluster also showed two cell types among CD1c^+^, CD123^+^, and CD141^+^ cells (**Figure 2E**). Per-cell biaxial AbSeq scatter plots revealed double-positive cells in the CD141 versus CD1c, CD141 versus CD123, and CD1c versus CD123 quadrants at 11.51%, 16.27%, and 0.33%, respectively, in the annotated DC compartment (**Figure 2F**). In the violin plots, CD141 and CD1c expression levels were similar between single-positive and double-positive clusters for the surface markers (**Figure 2G**). Similarly, the CD123^+^CD141^-^ and CD123^+^CD141^+^ clusters were single populations with high CD123 expression levels. In terms of CD11c expression, CD1c^-^CD141^+^ and CD1c^+^CD141^+^ clusters showed high CD11c expression. This analysis indicates that canonical markers were shared across multiple ADT-defined populations.

**Figure 2:**
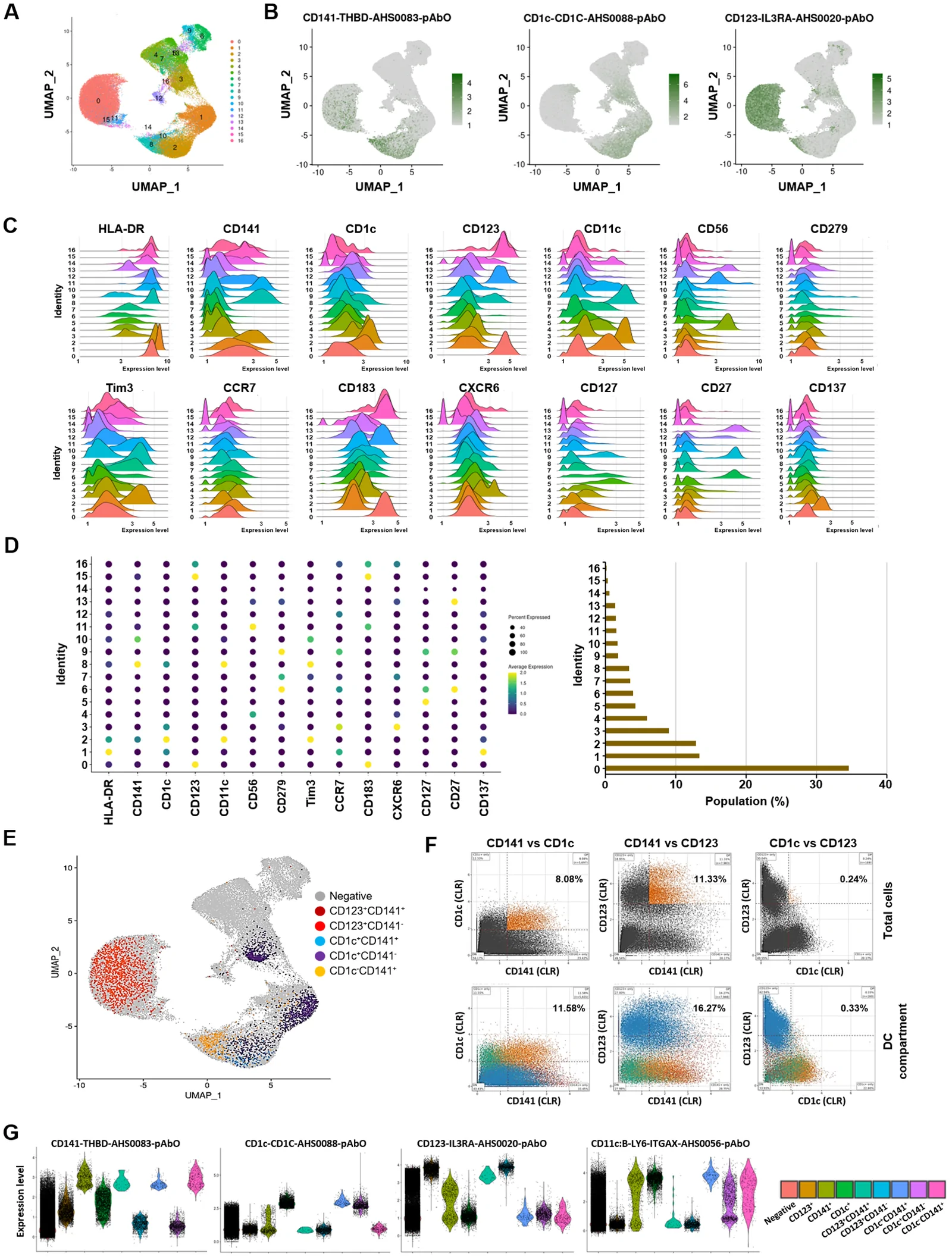
Clustering and surface marker expression of DC-enriched cells by AbSeq. **(A)** Clustering of cells in UMAP from 33 surface marker expression profiles of DC-enriched cells and cell populations in 17 clusters (C0-C16, color key at right). **(B)** UMAP of CD141, CD1c, and CD123 expression (AbSeq targets THBD, CD1C, and IL3RA). Green indicates increased expression; each marker is displayed on its own CLR-normalized scale. **(C)** Ridge plots showing surface marker expression across clusters. The y-axis (Identity) denotes clusters C0-C16. **(D)** Dot plots of surface marker expression within UMAP clusters (left), in which dot size indicates the percentage of cells expressing the marker and dot color indicates the average expression; the percentages of cells in each cluster (right). **(E)** UMAP of CD123, CD141, and CD1c expression in clusters. Increased expression is shown in colors. **(F)** Per-cell biaxial scatter plot and quadrant count of CD141 versus CD1c, CD141 versus CD123, and CD1c versus CD123 in total cells (upper row) and annotated DC compartment (lower row). Dots represent one cell. Both x and y axes are CLR-normalized ADT values obtained after within-cell normalization (margin = 2) of raw ADT counts. **(G)** Violin plots showing normalized AbSeq expression of representative surface markers.

### 3.4 Surface marker expression in heterogeneous subgroups of DC-enriched cells identified by single-cell multimodal profiling

scRNA-seq and AbSeq profiling of Lin-HLA-DR^+^ cells were simultaneously performed. We compared RNA-only, AbSeq-only, and joint WNN representations using the same 70,465 cells (**Supplementary Figure S5**). For a like-for-like comparison, the three representations were clustered using the same graph-clustering framework at resolution 0.3. Descriptive correspondence with the archived 26-population annotation was highest for WNN (Adjusted Rand Index (ARI)/Normalized Mutual Information (NMI), 0.894/0.919), followed by RNA-only (0.872/0.846) and AbSeq-only (0.776/0.687). Because the archived annotations were originally assigned with reference to the WNN cluster context, these values are presented as descriptive correspondence rather than as an independent classifier-performance test. The modality-specific views show that RNA and AbSeq contribute complementary information. RNA more clearly resolves transcriptomically distinct DC lineages, whereas AbSeq captures overlapping surface phenotypes; the joint WNN analysis integrates both information sources. The primary WNN analysis used resolution 0.6, while resolution 0.3 was used only for the matched modality comparison.

The unsupervised analysis identified 26 annotated populations with distinct immune cell expression profiles (**Figure 3A**). Among these, DC populations were further resolved by the surface expression of CD141, CD1c, and CD123, which were assigned to the cDC1, cDC2, and pDC lineages. These included three cDC1-lineage clusters (cDC1, cDC1-a, and cDC1-b), three cDC2-lineage clusters (cDC2, cDC2-a, and cDC2-b), and three pDC-lineage clusters (pDC, pDC-a, and pDC-b). In addition, the CD137-associated DC lineage was identified as distinct CD137-DC clusters (CD137-DC, CD137-DC-a, CD137-DC-b, and CD137-DC-c). The remaining clusters, which corresponded to non-DC populations not removed by lineage depletion, were annotated as CD56, CXCR6, CD127, CD27, CD4-T, CD8-T, and CD19 B populations. A UMAP with 26 clusters was used to visualize subtypes based on their transcriptional profiles (**Figure 3B**). The cDC1-a cluster separated from the cDC1 cluster and was closer to the cDC2 cluster in the UMAP. The percentages of clusters across different DC lineages were determined (**Figure 3C**). The pDC lineage had the highest frequency across the 26 clusters (35.7%), suggesting a dominant or heterogeneous pDC population. Among 6 clusters in the cDC1 and cDC2 lineages, the cDC2 cluster was the most abundant (12.2%). In the cDC1 lineage, the cDC1-b cluster (1.2 ± 0.7%) had a lower frequency than the cDC1-a cluster (2.4 ± 1.1%) (*p* < 0.05). UMAP revealed the spatial distribution of CD1c^+^, CD123^+^, CD141^+^ and other surface marker-positive cells in 26 clusters (**Figure 3D**; **Supplementary Figures S6A; S6B**). Next, the mean expression values of the highly expressed cell surface markers were scaled for each DC cluster (**Figure 3E**; **Supplementary Figures S6C and S6D**). cDC1 lineage expressing high levels of CD141 showed lower levels of CD1c than CD11c. The cDC1-a and cDC1-b clusters expressed the highest levels of CD11c among the surface markers, and these cells expressed higher levels of CD141 than CD1c. Although three clusters in the cDC2 lineage highly expressed CD1c, it was also expressed in the cDC1-a cluster and the CD137-DC and CXCR6^+^ lineages. In particular, Tim3 was highly expressed in the cDC1 and cDC2 lineages compared to the pDC and CD137-DC lineages. CD272, a B- and T-lymphocyte attenuator, was expressed in cDC1 and pDC lineages.

**Figure 3:**
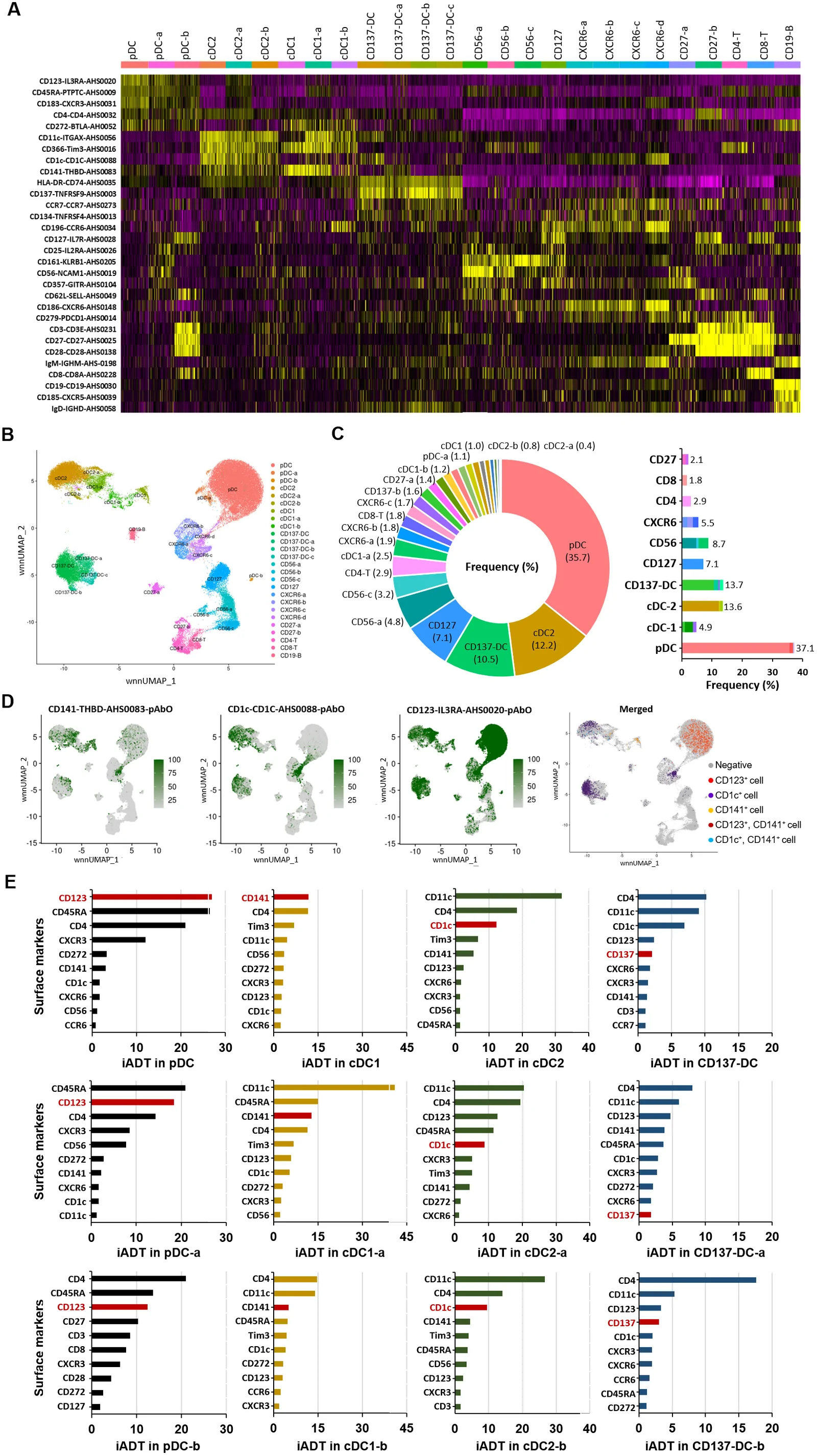
Clustering of Lin^-^HLA-DR^+^ cells by scRNA-Seq with AbSeq. **(A)** Heatmap showing AbSeq-derived surface protein expression across 26 annotated cell populations. Columns are individual cells grouped by population (color bar at top), and rows are the surface markers. **(B)** UMAP of the 26 clusters, which are labeled both in-plot and in the color key at right. **(C)** Proportions of cells assigned to subtypes in the left panel. Numbers indicate percentages of clusters. In the right panel, cluster frequencies are shown by major cell lineage, and different colors indicate the relative proportions of subgroups within each category. **(D)** UMAP showing expression of CD1c, CD141, and CD123 (green scale). The right panel, shown as Merged, displays cells classified as CD123^+^, CD1c^+^, CD141^+^, CD123^+^CD141^+^, or CD1c^+^CD141^+^. **(E)** Bar plots showing mean CLR-normalized AbSeq-derived surface marker expression (iADT, the batch-integrated ADT signal) for 12 DC-lineage clusters. Each cluster shows the 10 most highly expressed cell surface markers among 33 markers. The marker used to define each lineage is labeled in red.

### 3.5 Frequency of major clusters and differential surface marker expression in clinical stages and molecular subtypes

The frequency of major clusters was analyzed in the LN across different clinical stages of GC. The frequency of clusters in cDC1, cDC2, pDC, and CD137-DC lineages did not differ between EGC and AGC groups (**Figure 4A**; **Supplementary Figure S7A**). Among the molecular subtypes, we compared the frequencies of cDC2 and CD137-DCs between the chromosomal instability (CIN) and genomically stable (GS) molecular subtypes. cDC2 was significantly more frequent in CIN (n = 6) than in GS (n = 4) LNs (13.83 ± 5.92% vs 5.76 ± 3.50%; 2.40-fold; Welch’s t-test p = 0.027, Mann–Whitney p = 0.038; Hedges’ g = 1.42). CD137^+^ cells showed the same direction and a comparably large effect size (17.16 ± 10.03% vs 5.90 ± 5.52%; 2.91-fold; Hedges’ g = 1.18), but the difference did not reach statistical significance (Welch’s t-test p = 0.053, Mann–Whitney p = 0.067). Next, the expression levels of the surface markers were compared across the molecular subtypes of all 26 clusters (**Supplementary Figure S7B**). Average CD1c expression in cDC1-a and cDC1-b cells was higher in the GS subtype than in the CIN subtype (**Figure 4B**). However, CD141 levels were highest in the cDC1 and cDC1-a clusters in the GS and microsatellite instability (MSI) subtypes, respectively. CD123 expression in the three pDC clusters and cDC2-a cluster was higher in the CIN subtype than in the other subtypes (**Supplementary Figure S7C**). Tim3 expression in the cDC1 cluster was higher in the GS subtype than in the MSI subtype.

**Figure 4:**
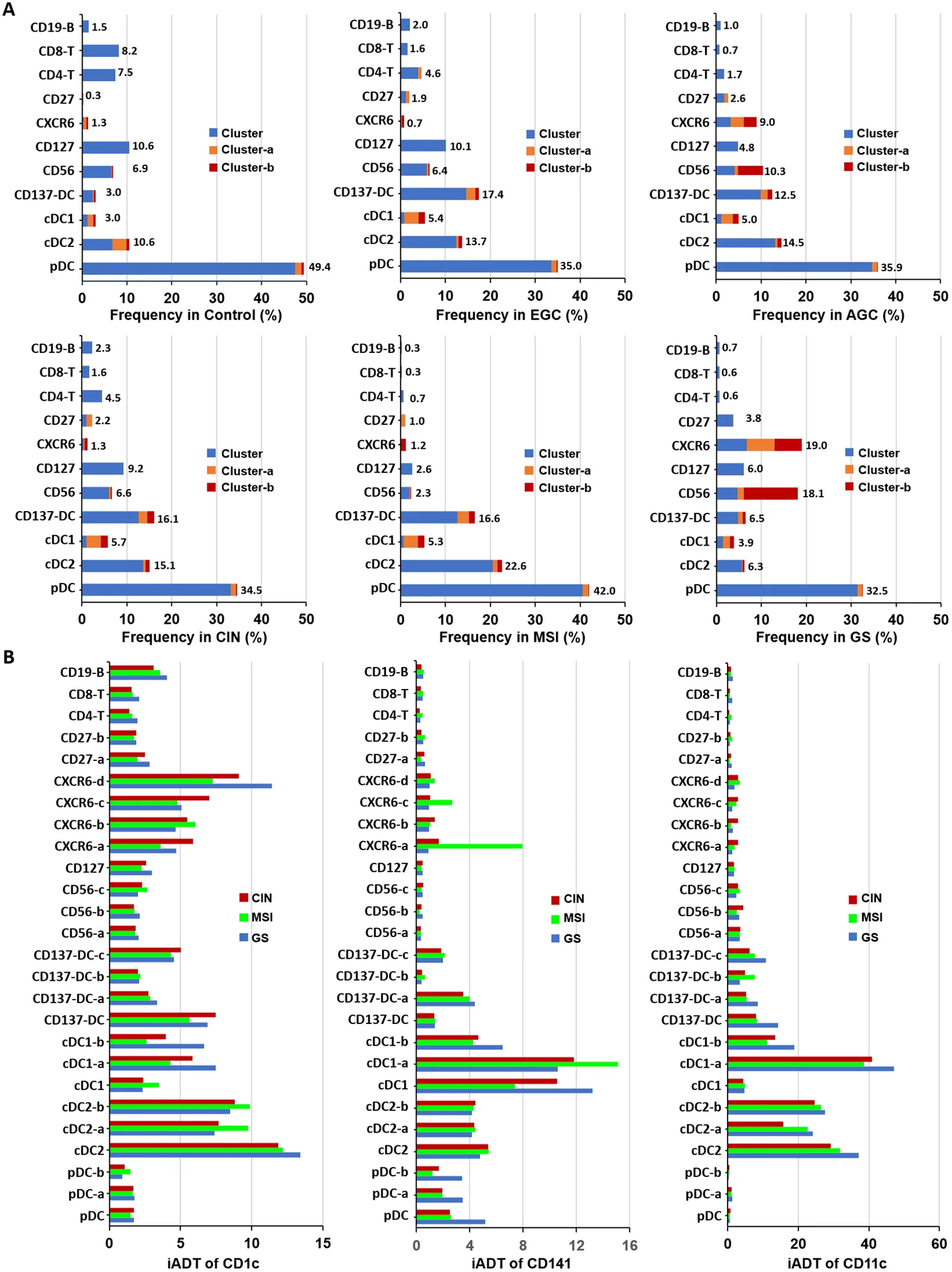
Frequency of major clusters and expression levels of major surface markers in clinical stages and molecular subtypes. **(A)** Frequency of the major clusters in total Lin^-^HLA-DR^+^ cells, shown separately for control LNs and for LNs grouped by clinical stage (EGC, AGC) and molecular subtype (CIN, MSI, GS). Different colors represent clusters in the related lineages. The value printed beside each bar is the summed frequency of that lineage. **(B)** Bar plots showing the mean integrated expression of selected surface markers by molecular subtypes. iADT indicates the CLR-normalized integrated ADT signal. EGC (n = 3), AGC (n = 8), CIN (n = 6), GS (n = 4), and MSI (n = 1). AGC, advanced gastric cancer; CIN, chromosomal instability; EGC, early gastric cancer; GS, genomically stable; MSI, microsatellite instability.

### 3.6 RNA expression in the cDC1 and cDC2 lineages

Differentially expressed genes characterizing each cluster were identified. Differentially expressed genes were defined as genes detected in more than 25% of cells within a cluster, with a log-fold change >0.25 and a Bonferroni-adjusted p-value < 0.05. To validate these categories, the transcriptional levels of the top 5–7 genes were visualized for each cluster (**Figure 5A**). Forty-eight marker genes were ranked according to their differential expression across the six clusters identified in cDC1 and cDC2 lineages (**Figure 5B**). *CLEC9A, CADM1*, and *XCR1* were expressed almost exclusively in the cDC1 cluster, which are previously known cDC1-related genes (5). Other cDC1-related genes, including *CPNE3, CLNK, and C1orf54*, were also identified. In the cDC1 lineage, *XCR1 and CADM1* transcripts identified cDC1; *SIGLEC6* and *IL22RA2* transcripts identified cDC1-a; and *HMGB2* and *MKI67* identified cDC1-b. *GZMB*, *DPYD*, and *CLEC10A* were expressed almost exclusively in the cDC2 lineage, but only the cDC2-a cluster showed high *GZMB* expression. All clusters in the cDC1 and cDC2 lineages expressed *ITGAX* (CD11c), with higher expression in the cDC1-a cluster than in the cDC1-b and cDC1 clusters. *S100B* was expressed in the cDC1 and cDC1-b clusters, but not detected in the cDC1-a cluster. Moreover, *CLNK, CLEC9A, C1orf54, S100B, FABP5, and ANPEP* were identified in the cDC1 and cDC1-b clusters but not detected in the cDC1-a cluster. *THBD* was expressed in the cDC1, cDC1-a, and cDC2-a clusters. The cDC2 lineage and cDC1-a clusters expressed *IL-1B*, *CD52*, and *PTGS2*, but the cDC1 cluster showed no detectable transcripts. These gene expression results indicate that cDC1-b shared more of the selected cDC1-associated markers than cDC1-a. Gene expression across these clusters in the different clinical stages and molecular subtypes was evaluated (**Supplementary Figure S8A**). *CLEC9A* expression did not differ across clusters of the cDC1 and cDC2 lineages at different stages and molecular types (**Figure 5C**). IRF8 transcripts were detected in the cDC1 and cDC2-a clusters across the three molecular subtypes but were not detected in the cDC2 and cDC2-b clusters. EGC and AGC showed lower IRF8 expression in the cDC2 and cDC2-b subclusters than the control samples. *IDO1* expression in the three clusters of the cDC2 lineage and in cDC1-a cells was higher in the GS subtype (**Supplementary Figure S8B**).

**Figure 5:**
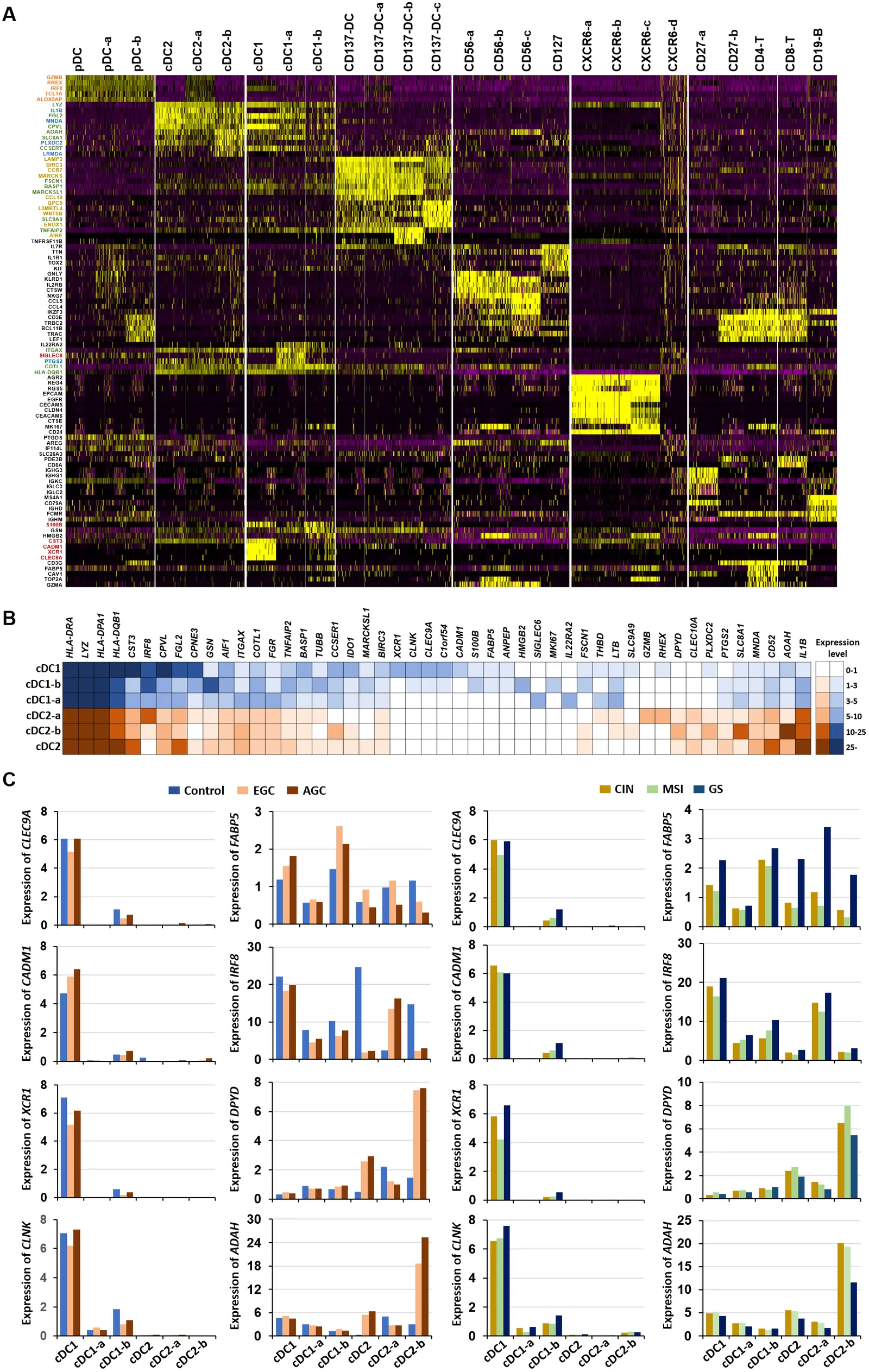
Clustering analysis of differentially expressed genes. **(A)** Validation of clustering analysis from scRNA-Seq and AbSeq data. Heatmaps of similar gene expression patterns within clusters. The top five to seven differentially expressed genes per cluster are shown across the 26 annotated populations; columns are individual cells grouped by population, and scaled expression runs from purple (low) to yellow (high). **(B)** Heatmap of gene expression patterns across cDC1 and cDC2 lineages. Forty-eight marker genes are ranked by differential expression across the six cDC1- and cDC2-lineage clusters. Color intensity encodes the binned mean expression level, with the blue and orange scales denoting the cDC1 and cDC2 lineages, respectively. **(C)** Gene expression across different GC stages and molecular subtypes. Mean expression of 8 genes is shown for the six cDC1 and cDC2 clusters. AGC, advanced gastric cancer; CIN, chromosomal instability; EGC, early gastric cancer; GS, genomically stable; MSI, microsatellite instability.

### 3.7 RNA expression in the clusters of pDC and CD137-DC lineages

*IRF8, RHEX, GZMB, ALOX5AP*, *PTGDS*, and *IRF7* were highly expressed in pDC clusters (**Figure 6A**). Among these genes, *PTGDS* expression was higher in MSI and GS subtypes than in the CIN subtype (**Figure 6B**). In CD137-DCs, *BIRC3*, *LAMP3*, *FSCN1*, *MARCKSL1*, and *CCR7* were highly expressed; they are strongly associated with a specific subset of mature, migratory, and activated DCs, often referred to as mature DCs enriched in immunoregulatory molecules (**Figure 6C**) (26). These genes were low or undetectable in the cDC1, cDC2, and pDC lineages. Conversely, CD137-DCs lacked detectable expression of the lineage-defining genes of the cDC1 (*CLEC9A, XCR1*), cDC2 (*CLEC10A*), and pDC (*IRF8*) lineages, supporting their identity as a distinct population.

**Figure 6:**
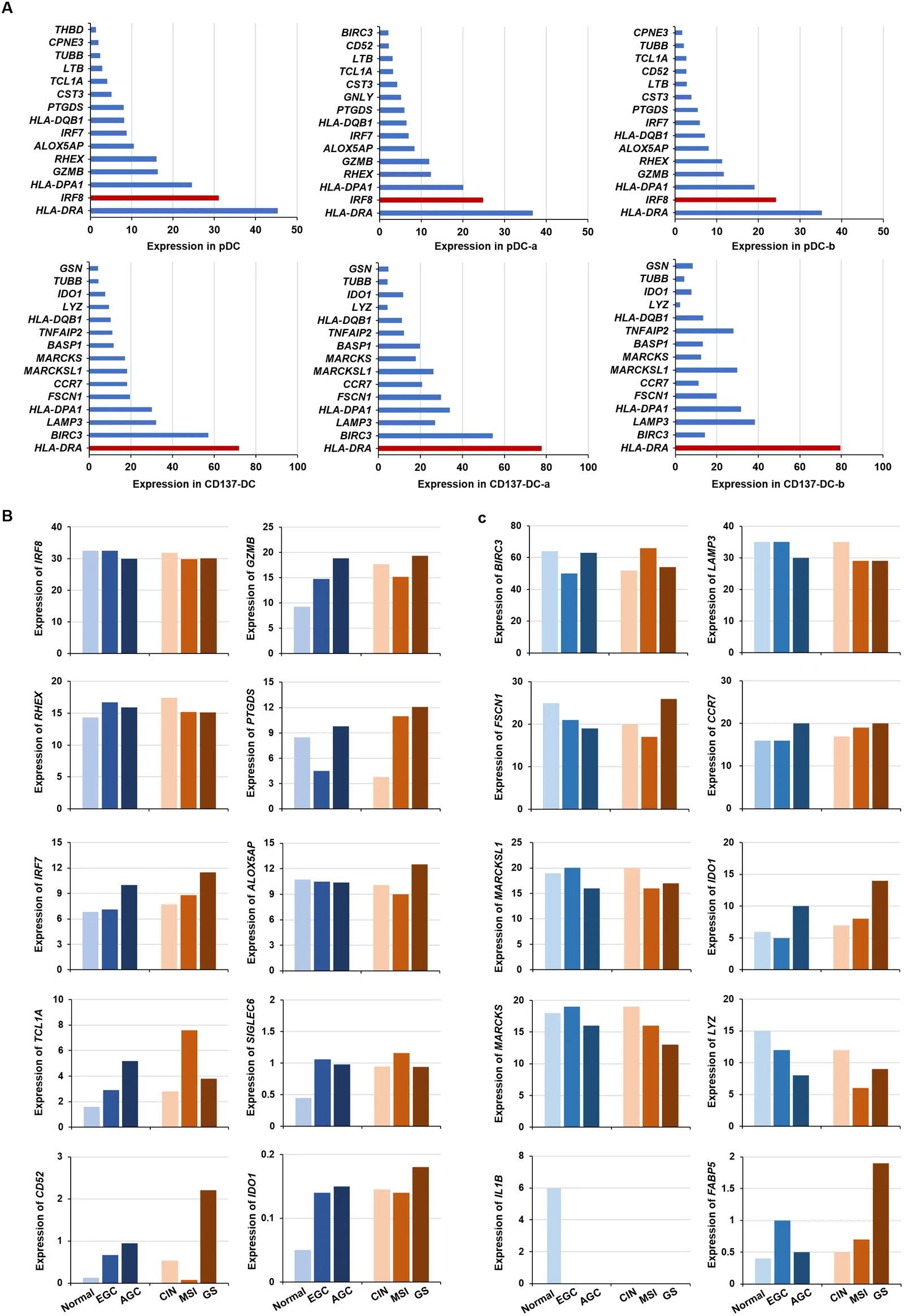
Differential gene expression patterns in pDC and CD137-DC lineages. **(A)** Expression levels of major genes in the clusters of pDC and CD137-DC lineages. The 15 most highly expressed genes are shown for each of the six clusters, ranked by mean normalized RNA expression level (x-axis). Red bars highlight *IRF8* in the pDC clusters and *HLA-DRA* in the CD137-DC clusters. **(B, C)** Expression of genes in the pDC **(B)** and CD137-DC **(C)** clusters in clinical stages and molecular subtypes. Ten genes are shown per lineage. Blue bars denote clinical stage (normal, EGC, and AGC), and orange bars denote molecular subtype (CIN, MSI, and GS). Note that the y-axis scale differs between genes. AGC, advanced gastric cancer; CIN, chromosomal instability; EGC, early gastric cancer; GS, genomically stable; MSI, microsatellite instability.

### 3.8 Detection patterns of selected *TLR* and transcription factor transcripts

*TLR* transcripts can be detected in each DC subtype. *TLR2* expression was detected in the cDC2 lineage and cDC1 cluster, and *TLR4* expression was detected in cDC1-a, cDC1-b, and cDC2 clusters (**Figure 7A**). *TLR3* expression was detected in the CD137-DC lineage, but *TLR7* expression was detected in the pDC lineage and cDC2-a cluster. However, very low levels of *TLR7* and undetectable levels of *TLR4* were observed in the cDC1 lineage.

**Figure 7:**
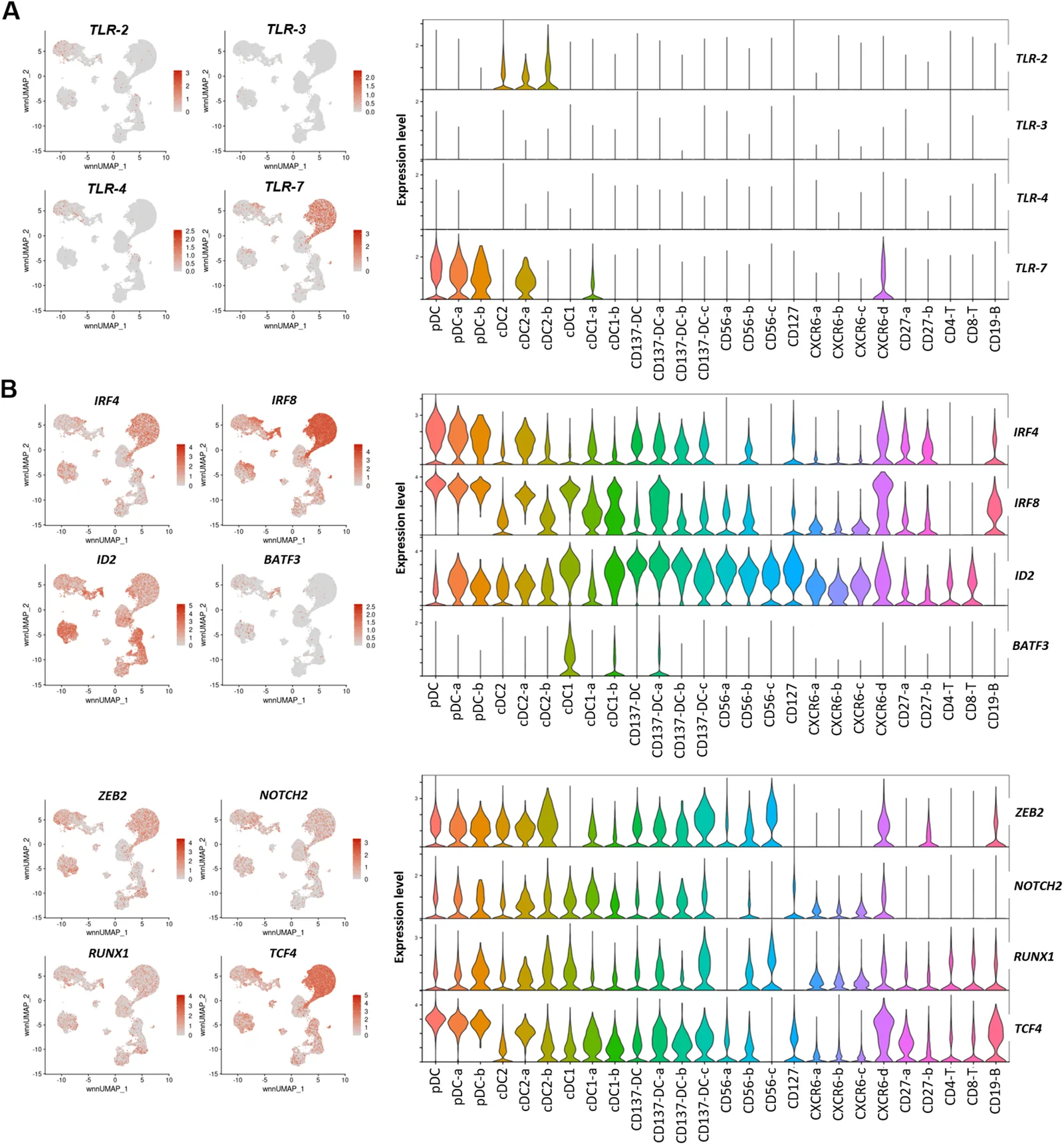
Gene expression of TLRs and transcription factors. **(A)** UMAP and violin plots showing TLR gene expression across 26 clusters. *TLR2*, *TLR3*, *TLR4*, and *TLR7* are shown as WNN-UMAP overlays (left) and as violin plots across the 26 annotated populations (right). **(B)** UMAP and violin plots showing the expression of key transcription factor genes. Color intensity on the UMAP reflects normalized RNA expression, and gray indicates no detectable expression. TLR, toll-like receptor.

Lineage-defining transcription factor expression is conserved between mouse and human cDC1 cells (27). Specific transcription factor genes were overlaid onto UMAP to visualize the distribution of selected lineage-associated transcription factor transcripts across clusters; violin plots show their expression levels (**Figure 7B**). *BATF3* expression was primarily positive in the cDC1 cluster and, to a lesser extent, in the cDC1-b and CD137-DC-a clusters, but was nearly below the detection threshold in the cDC2 lineage. *IRF4* is considered a key transcription factor for cDC2 cells (28). Violin plots confirmed that *IRF4* was found mainly in the pDC and cDC2-a clusters. *TCF4*, the primary driver of pDC development that activates *IRF8* (29), was highly expressed in the pDC lineage and cDC2-a cluster. *IRF8* expression was high in pDC, cDC1, and cDC2-a clusters. High *ID2* expression was detected in the cDC1 and cDC1-b clusters and the CD137-DC lineage, but expression was very low in the cDC2-a cluster. *ZEB2* is essential for pDC and cDC2 development and identity (30). *ZEB2* was positive in the cDC1-a and cDC1-b clusters, as well as in the cDC2 lineage, but was not detected in the cDC1 cluster. *NOTCH2* and *RUNX1* expression was high in the cDC1 lineage and cDC2-a clusters, although *NOTCH2* is essential for the differentiation of a specific subset of CD11b^+^ cDC2 cells, and *RUNX1* is vital for maintaining cDC2 cells (31, 32).

### 3.9 Distribution of surface marker-positive cells in immunohistochemically stained LNs

The LNs were immunostained to ascertain the distribution of surface marker-positive cells. CD141 immunoreactivity was concentrated around vessels in the mantle zone and medulla (**Figure 8A**; **Supplementary Figure S9**). No clear differences in this distribution pattern were observed across tumor stages and molecular subtypes. Because CD141/THBD is also expressed by endothelial cells and monocytes (33, 34), CD141 staining alone cannot be attributed to cDC1; CLEC9A was therefore used as the cDC1-specific marker for *in situ* localization. In control samples, CLEC9A^+^ cells were broadly distributed within the deep paracortex, but in LNs from patients with cancer, these cells were less frequent and more sparsely distributed in the same region. CD1c^+^ cells were predominantly located in proximity to high endothelial venules, as well as within the paracortical and interfollicular areas. The MSI subtype showed a greater presence of CD1c^+^ cells surrounding endothelial venules than the control sample. CD137^+^ cells were primarily confined to the lymphoid follicles of control LNs. In LNs from patients with cancer, however, CD137^+^ cells were more frequently observed in interfollicular regions. CLEC10A^+^ and S100B^+^ cells were detected mainly in the paracortical and interfollicular regions. Although overall cell density did not differ between LNs from controls and patients with cancer, cell distribution in cancer samples appeared more dispersed. S100B^+^ cells exhibited large dendritic morphology.

**Figure 8:**
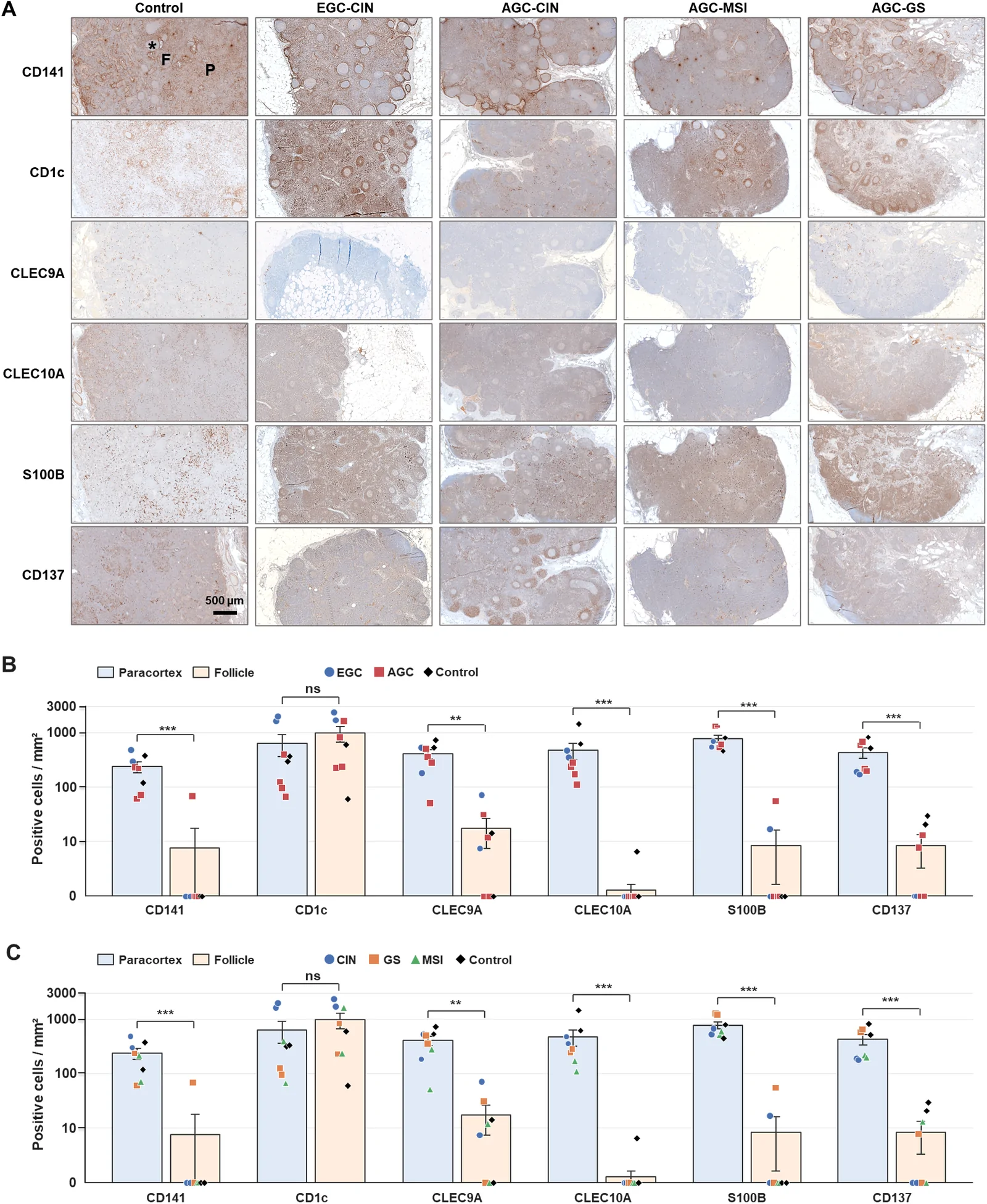
Immunohistochemical localization and quantification of surface marker expression in LNs. **(A)** Paraffin-embedded LN sections were stained for surface marker expression by immunohistochemistry (x100). The scale bar (500 µm) applies to all panels. The control is a non-neoplastic LN; the remaining columns are LNs from GC patients. Markers on the CD141 panel of the control column are as follows: F, follicle; P, paracortex; asterisk, germinal center. **(B, C)** Density of marker-positive cells in the paracortex and in lymphoid follicles. Values are given as positive cells per mm^2^ for CD141, CD1c, CLEC9A, CLEC10A, S100B and CD137; each symbol represents one LN. Bars represent the mean ± SEM of eight fields per compartment. The vertical axis is a symmetric logarithmic scale. Both panels show the same data, colored by clinical stage in **(B)** and by molecular subtype in **(C)**. ***adjusted p < 0.005, **adjusted p < 0.01, ns, not significant (Mann-Whitney U test with Benjamini-Hochberg correction). AGC, advanced gastric cancer; CIN, chromosomal instability; EGC, early gastric cancer; GS, genomically stable; LN, lymph node; MSI, microsatellite instability.

Next, positive cell densities were measured in the paracortex and in lymphoid follicles (**Figures 8B, C**). CD141-positive cells were detected in the paracortex (226.4 versus 0.0 cells/mm²; adjusted p = 0.0013). Capillaries within the parenchyma may not have been entirely excluded from the counts. CD1c-positive cells were as numerous within follicles as in the paracortex (718.8 versus 332.1 cells/mm², p = 0.51). Within the follicle, they occupied the mantle zone and spared the germinal center. CD1c alone therefore cannot identify cDC2 in tissue sections. CLEC9A labeled far fewer cells, singly dispersed in the deep paracortex and virtually absent from follicles (438.5 versus 10.4 cells/mm²; adjusted p = 0.0016). CLEC10A showed a similar paracortical distribution (321.4 versus 0.0 cells/mm²; adjusted p = 0.0013). S100B-labeled cells were distributed through the deep paracortex and interfollicular regions and gave the highest density of the six antibodies (median 647.1 cells/mm² in paracortex versus 0.0 in follicles; adjusted p = 0.0013). CD137-positive cells were distributed in the paracortex. Germinal centers were negative in the control LN and in all three tumor-draining LNs, including follicles with well-formed reactive centers (374.4 versus 4.5 cells/mm²; adjusted p = 0.0013). Overall, these findings indicate spatial reorganization of DC–associated populations in tumor-draining LNs.

### 3.10 Limitations

In this study, RNA and ADT data were integrated across the four batches using rPCA-based batch correction, but clinical stage and molecular subtype were not fully balanced across batches, and multiple LN specimens were obtained from some patients. In particular, the MSI category contained a single sample from one patient in DC-set01, so patient, subtype, and run effects cannot be separated for MSI. Therefore, residual confounding between biological groups and sequencing batches cannot be completely excluded. Comparisons among clinical and molecular subgroups should accordingly be considered exploratory. All LN specimens were enzymatically dissociated for 30 min at 37 °C, and an enzyme-free matched control was not evaluated because enzymatic digestion was required to obtain sufficient cell yield and DC recovery from LN tissue. Warm enzymatic dissociation may induce immediate-early and heat-shock transcriptional programs and may alter surface-epitope detection in a cell-type-dependent manner. As a *post hoc* assessment, we evaluated *HSPA1A*, *HSPA1B*, and a 15-gene heat-shock/chaperone panel across all 70,465 cells. These genes showed broadly overlapping expression across the 26 annotated populations rather than being restricted to a single population. In additional sensitivity analyses, the principal cDC1, cDC2, pDC, and mature CD137-DC programs remained detectable in the lowest stress-score quartiles, and reconstruction after removal of predefined stress-response genes retained substantial agreement with the archived annotations (ARI, 0.745 for WNN and 0.877 for RNA-only). Correlations between the heat-shock score and nine CD137-DC maturation-associated genes were weak (Spearman ρ, −0.064 to 0.111). **Supplementary Figure S10** shows the distribution of heat-shock and chaperone transcripts across all annotated populations. These analyses do not rule out dissociation-induced effects. Rather, they indicate that the measured stress-response features alone did not generate the major DC programs and population structure.

## 4 Discussion

In this study, single-cell clustering resolution was improved by distinguishing closely related DCs using integrated surface marker and RNA expression data from Lin^-^HLA-DR^+^ cells. Flow cytometry and ADT analyses could not distinguish cDC1 from cDC2 based on surface expression of CD141 and CD1c. In mice, some mature cDC1 cells express surface features characteristic of cDC2, suggesting that multiple cDCs can co-express lineage-overlapping features (35). CD8^+^XCR1^-^ mouse DCs express lineage-mixed gene signatures of cDC1, cDC2, and pDCs (36).

Human cDC1s are identified by CD141 expression in LNs. Gene and surface marker expression of XCR1 was exclusively detected in the cDC1 cluster. XCL1, produced by cancer cells, is a chemotactic ligand for XCR1 on cDC1 cells (37). PD-L1 was primarily detected in the XCR1^+^CD141^+^CD1c^-^ population. Mayoux et al. suggested that PD-L1 in DCs is a superior predictor of response to anti-PD-L1 therapy compared to tumor cell PD-L1, although suppressive DCs express high levels of PD-L1 (38). In addition, the cDC1 lineage expressed TIM-3 and CD272. TIM-3 inhibits the endocytosis of extracellular DNA and activation of the cytoplasmic DNA-sensing pathway in cDC1 cells, thereby limiting antitumor immunity (39). CD272, expressed on specific DC subsets, plays an inhibitory role in immune regulation by promoting regulatory T cell differentiation and reinforcing immunosuppressive networks (40).

CD1c is the primary surface marker for identifying human blood cDC2 cells. However, CD1c expression was detected in CD141^+^ cells and other cell types, including CD137-DC, CXCR6^+^, and CD19^+^ cells. *ITGAM* (CD11b), a key transcript of mouse cDC2, was either undetectable or very low in the cDC2 lineage. CD14 depletion to isolate Lin^-^HLA-DR^+^ cells likely eliminated the CD1c^+^CD14^+^ DC3 subset. ADT analysis shows that the CD14 surface marker was undetectable in the Lin^-^HLA-DR^+^ cells. CD88^-^CD1c^+^CD5^-^ DC3s can be divided into three subsets: CD5^-^CD163^-^ cells, CD163^+^CD14^-^ cells, and CD1c^low^CD163^+^CD14^+^ cells (15, 41). CD1c^+^CD163^+^CD14^-^ DC3s may exist in the cDC2 population, but the *CD163* transcript was not detected in the cDC2 lineages, although CD14^-^ DC3s may exist in a cDC2 lineage.

CD137-DCs were identified as distinct from the cDC1 and cDC2 lineages in our dataset. CD137-DCs exhibited some maturation characteristics. Transcriptome analyses have confirmed that human follicular DCs express genes associated with T cell regulation, specifically HLA-DRA (42). CD137 is also expressed by follicular helper T cells and follicular DCs (FDCs) (43, 44); however, FDCs are of mesenchymal rather than hematopoietic origin and were excluded from our Lin^-^HLA-DR^+^ single-cell dataset. In immunostained sections, CD137^+^ cells within follicles likely correspond to follicular helper T cells and FDCs, but the interfollicular CD137^+^ cells increased in LNs, which is more consistent with the CD137^+^ mature and migratory DC-like population identified by multimodal profiling. CD137-DCs highly expressed genes related to maturation and migration, such as *BIRC3*, *LAMP3*, *FSCN1*, and *CCR7*, suggesting that they may be activated or immunomodulatory DCs (45). CD137 is expressed on the surface of T cells, natural killer cells, and DCs (46). We asked whether the same signature appeared in bulk tissue by using *TNFRSF9*, the gene encoding CD137, in TCGA-STAD (n = 401) (**Supplementary Figure S11**). *TNFRSF9* co-varied with the genes that define the CD137-DC clusters, such as *LAMP3*, *HLA-DRA*, *CCR7*, and *BIRC3* (Spearman rho 0.61, 0.61, 0.60, and 0.53, respectively). Overall survival did not differ above and below the median (log-rank p = 0.16). The immune context was more informative: *TNFRSF9* increased with overall immune infiltration (ESTIMATE ImmuneScore, rho = 0.71) and, within the immune compartment, with activated CD4 memory T cells, follicular helper T cells, CD8 T cells, and M1 macrophages, and inversely with resting CD4 memory T cells and monocytes. It showed no relation to the estimated DC fraction. In bulk tissue, therefore, *TNFRSF9* marks an inflamed, T-cell-activated tumor rather than a DC population. These results suggest that identifying a distinctive CD137-DC subtype requires single-cell data.

More than 48 genes were differentially expressed in the six clusters of the cDC1 and cDC2 lineages. Human blood samples show that CD1c^+^ cDCs express *CLEC10A, CD1c*, and *ZEB2*, and CD141^+^ cDCs express *CLEC9A, IDO1*, and *IRF8* (47). *CADM1* is highly expressed on cutaneous CD141^+^ DCs, which are migratory, but LN-resident cDC1s often show low *CADM1* expression, compared to high expression of *XCR1* (48, 49). This study showed expression of both *XCR1* and *CADM1* in the cDC1 cluster, but undetectable in the cDC1-a and cDC1-b clusters. *BATF3* and *ID2* synergize to promote *IRF8*-directed cDC1 development and enhance lineage commitment (50). The acquisition of a cDC1 phenotype depends on *BATF3*, and CD8^+^ cDCs in *BATF3*-depleted mice co-express surface molecules characteristic of both cDC1 and cDC2 lineages (35, 51). In this analysis, *BATF3* was expressed exclusively in the cDC1 cluster, with low levels in the cDC1-b and CD137-DC clusters but undetectable in the cDC1-a cluster. Thus, the cDC1-b cluster may be closer to the cDC1 cluster than to the cDC1-a cluster. *ZEB2* loss increases ID2 expression and subsequently shifts cells toward the cDC1 lineage (30). *ZEB2* was not detected in the cDC1 cluster but was positive in the cDC1-a and cDC1-b clusters, suggesting that these clusters may downregulate *ID2* expression, resulting in CD1c^+^CD141^+^ cells. cDC2 differentiation is driven by the transcription factors *IRF4, ID2, ZEB2*, and *NOTCH2/KLF4*, and this subtype is identified by the expression of CD1c and CLEC10A in humans (52, 53). *ID2* is typically absent or repressed in mature, steady-state pDCs to allow the activity of E2-2 (TCF4), which is required for pDC identity (53). *ID2* expression was higher in cDC1 and cDC1-b clusters than in clusters of cDC2 and pDC lineages, which expressed moderate levels of *ID2*. *S100B* expression was observed in the cDC1 and cDC1-b clusters but not in the clusters of cDC2 and pDC lineages. In the immunohistochemical analysis, S100B^+^ cells exhibited large dendritic morphology. Histologically, S-100B is expressed particularly in interdigitating DCs and follicular DCs in lymphoid tissue and Langerhans cells in cutaneous tissue (54, 55). Interdigitating DCs, which are recognized as resident cDC1 and cDC2 cells, are large dendritic cells that form complex networks with several small T lymphocytes and are found in the paracortex. Thus, S100B may be a candidate marker warranting further validation.

tDCs exhibit a hybrid transcriptome and markers associated with both pDCs and cDC2 (7). In this study, *GZMB* was highly detected, and *IRF4* and *TCF4* were mainly expressed in the pDC and cDC2-a clusters. Tcf4 is essential for tDC development (7). CD123 expression in ADT analysis was higher in the cDC2-a cluster than in the cDC2 and cDC2-b clusters. Thus, we speculate that cDC2-a may be a subtype similar to tDCs in LN. However, no single cellular intermediate acts as a transition state between cDC1 and cDC2 under steady-state conditions. CD141^+^CD1c^+^ human pre-conventional DCs are found natively in the bone marrow, blood, or cord blood under steady-state conditions (56). cDC1 and cDC2-a clusters share surface phenotypes and transcriptomes, and cDC1-a profiles overlap with those of cDC2. Thus, a distinct CD141^+^CD1c^+^ subtype may represent a transitional phenotype between cDC1 and cDC2 in LNs.

The role of LN DCs in GC progression and prognosis remains uninvestigated. Accurate identification of DCs in LNs can help define DC subtypes in cancer tissues. An investigation of the relationship between GC subtypes and prognosis using gene expression data suggested that patients with MSI and CIN subtypes had better overall survival than those with the GS subtype (57). MSI-subtype cancers have been shown to respond more strongly to immune checkpoint blockade (58), suggesting that this high response rate is attributable to increased immunogenicity, thereby making these tumors more recognizable to the immune system. ADT analysis showed that clusters in cDC1, cDC2, and pDC lineages differentially expressed CXCR3, CXCR6, CCR6, and CCR7 on their cell surfaces. However, their expression did not differ across GC molecular subtypes.

In summary, analysis of DC surface markers from human LNs failed to identify classical cDC1, cDC2, or pDC subtypes because multiple specific markers were detected across DC lineages. Cluster analysis of Lin^-^HLA-DR^+^ LN cells revealed heterogeneous subtypes of cDC1, cDC2, pDC, and classical DCs. We also identified other DC subtypes, such as CD137-DCs, that expressed high levels of HLA-DR, CD1c, and other markers and genes. CD137-DCs identified in LNs highly express genes related to maturation and migration, suggesting they may be activated or immunomodulatory DCs. Precise identification of DC subtypes using AbSeq and scRNA-Seq could predict response to therapy, such as immune checkpoint inhibitors and chemotherapy, because different subtypes may exhibit distinct expression patterns in tumor-draining DCs.

## Data Availability

The processed single-cell data generated in this study have been deposited in the NCBI Gene Expression Omnibus (GEO) under accession number GSE348412 (https://www.ncbi.nlm.nih.gov/geo/query/acc.cgi?acc=GSE348412). Raw sequencing data are not publicly available to protect patient privacy.
