# Supplementary material for: Identification of heterogeneous subtypes of human dendritic cells from the lymph nodes of patients with gastric cancer
Source: Front Immunol. 2026 Oct 2;17:1912586. doi: 10.3389/fimmu.2026.1912586 (PMC13672212; doi:10.3389/fimmu.2026.1912586)
Supplement: Supplementary file 1 [file DataSheet1.pdf]

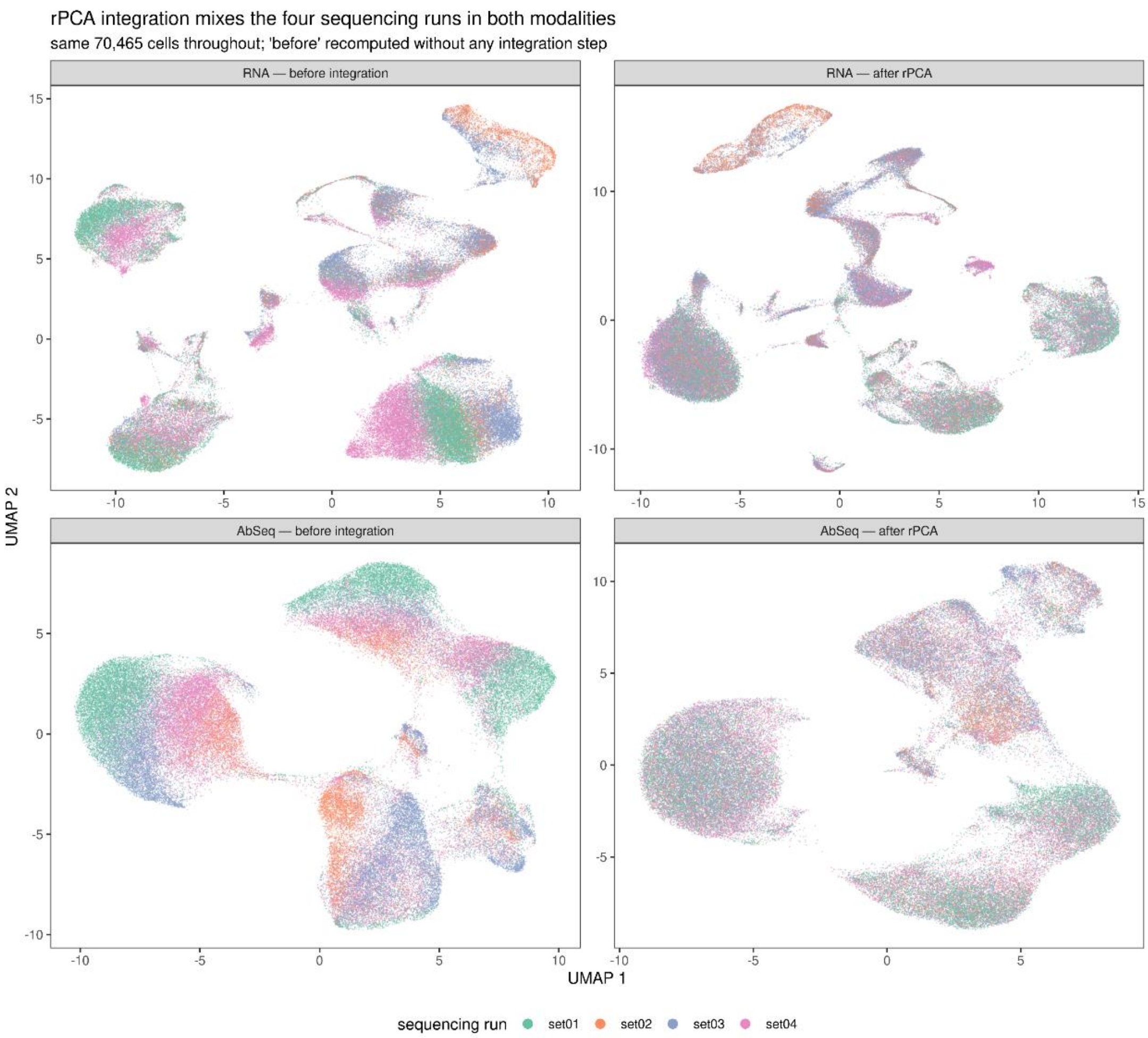

Supplementary Figure S1. Reduction of run-associated structure after rPCA integration of the RNA and AbSeq modalities. UMAP visualizations show the same 70,465 cells before integration (left) and after reciprocal PCA (rPCA)-based anchor integration (right) for RNA (top) and AbSeq (bottom). Cells are colored by processing/sequencing run (DC-set01–DC-set04). The unintegrated embeddings were recomputed without an integration step, whereas RNA and AbSeq were integrated separately to obtain the post-integration embeddings. The increased intermixing of run colors after integration is consistent with reduced run-associated structure.

ADT only: 0.3-0.9; selected 0.4

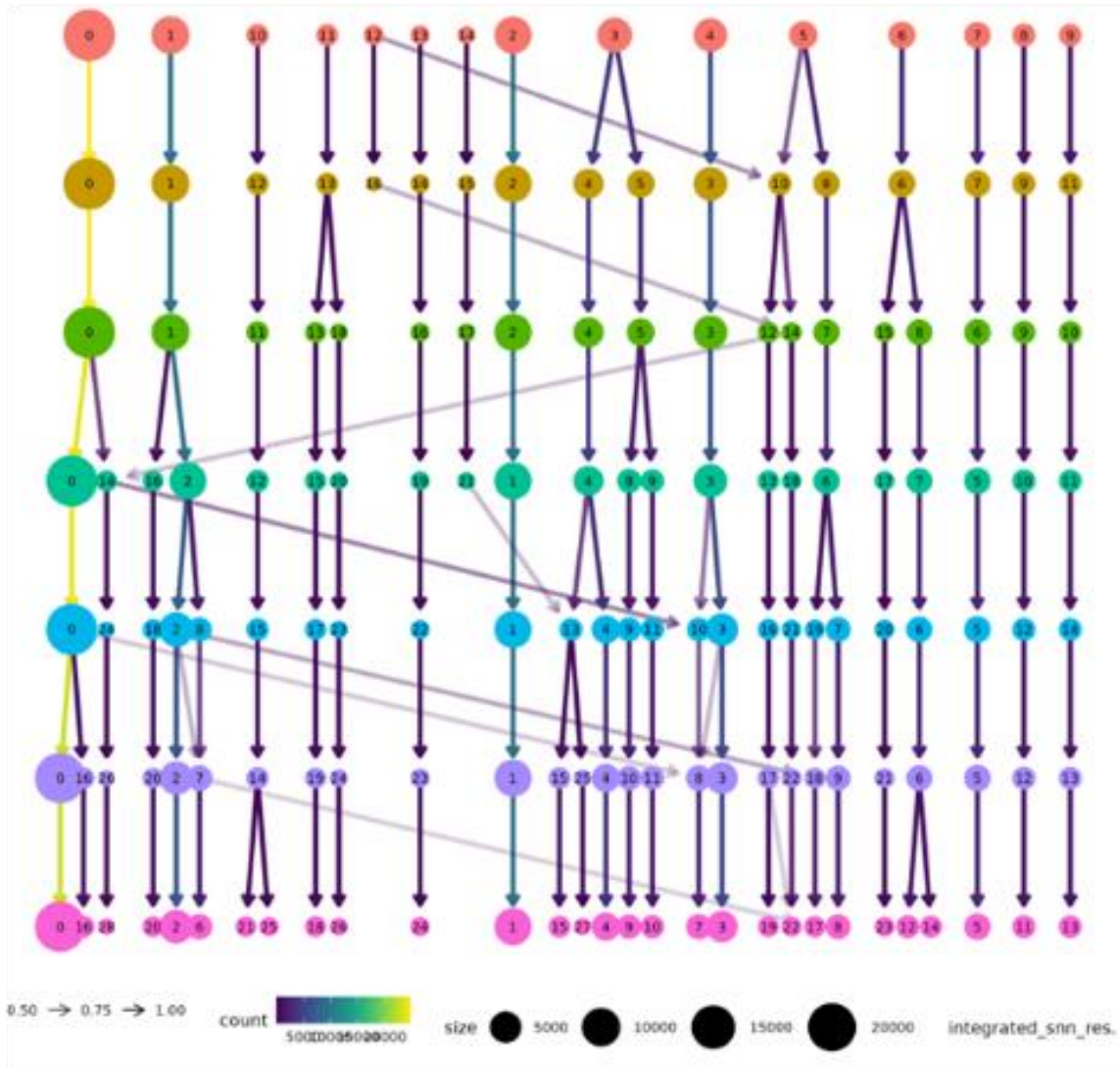

RNA only: 0.3-0.9; selected 0.5

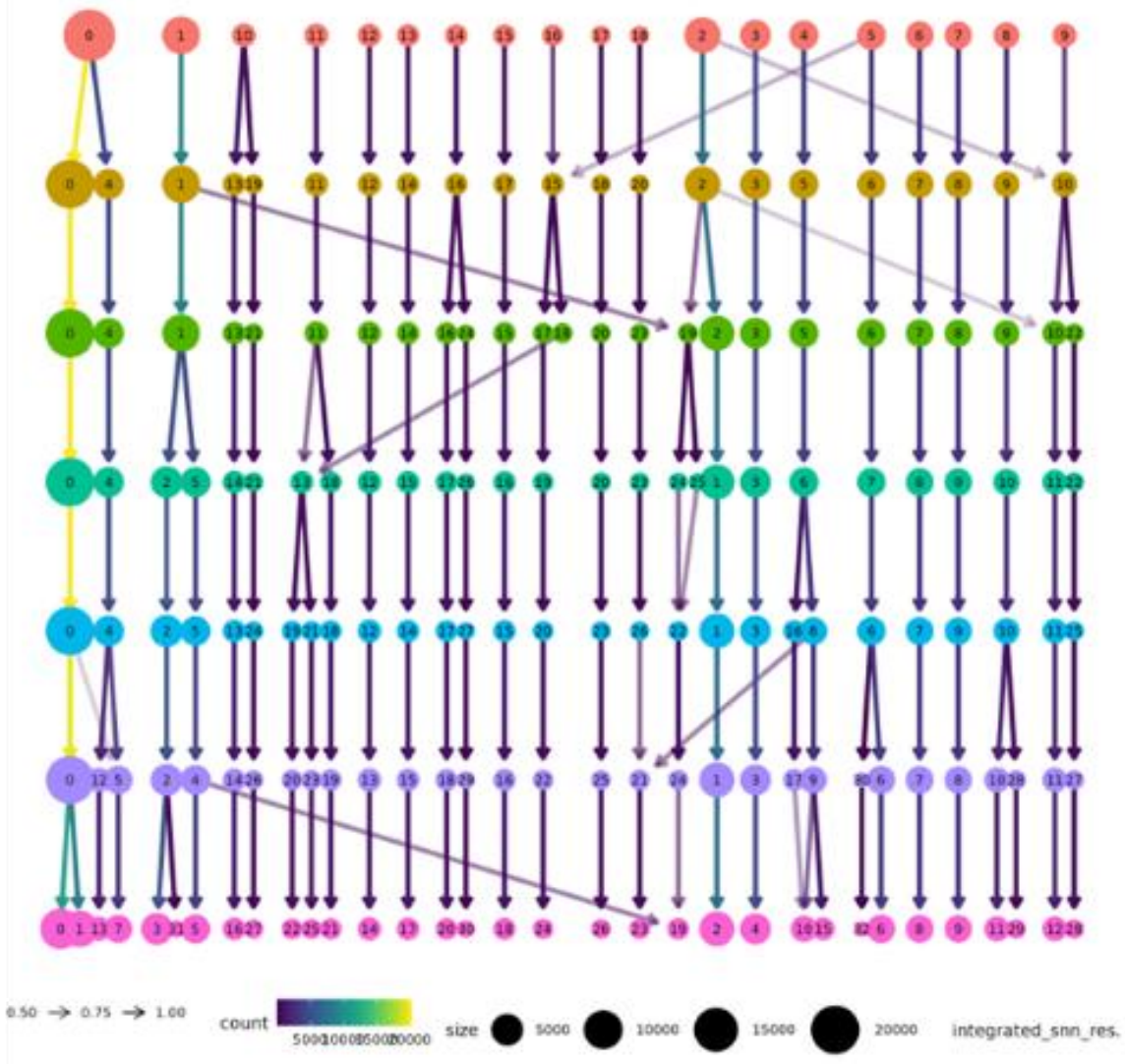

WNN: 0.3-1; selected 0.6

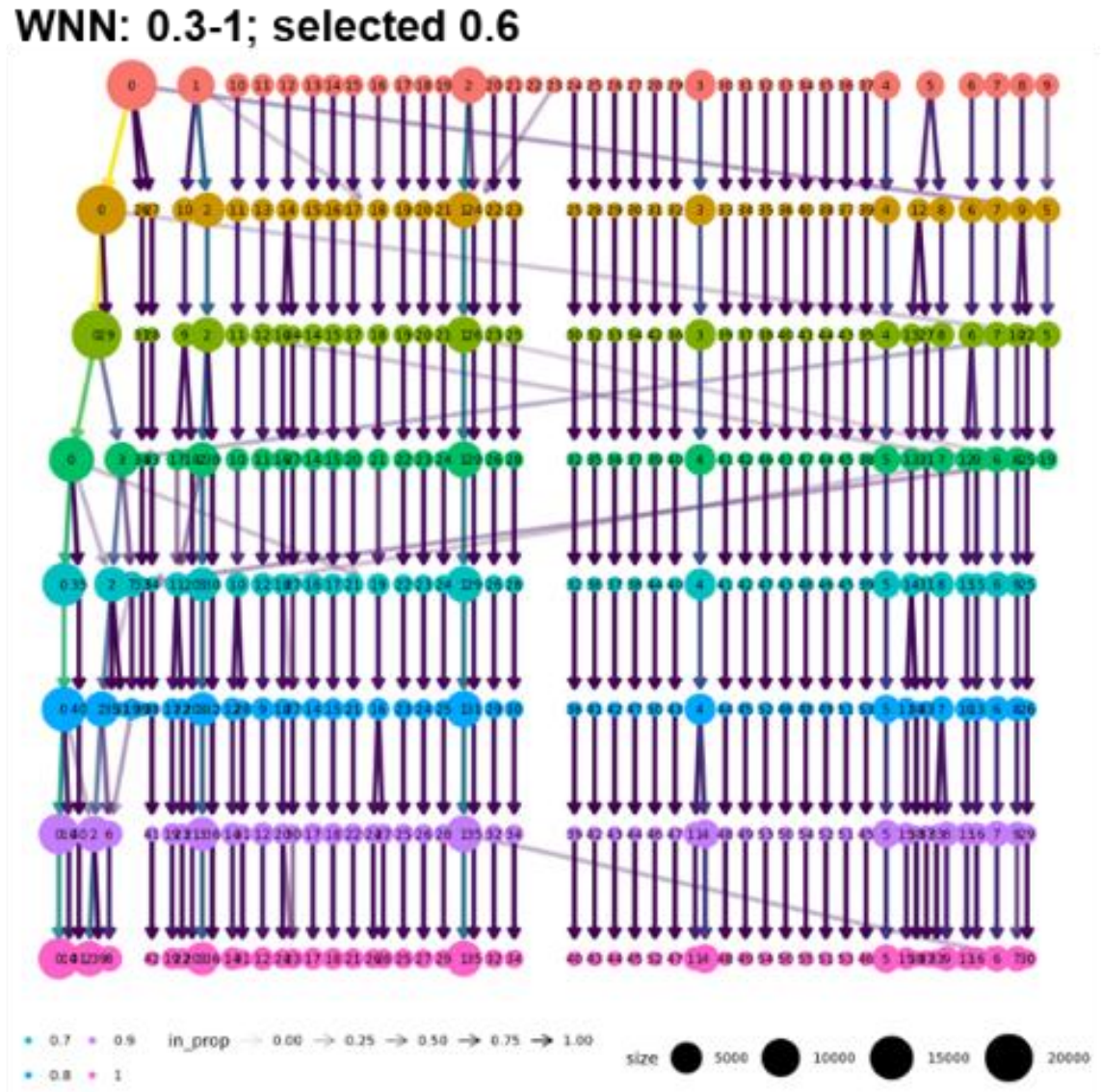

Supplementary Figure S2. Clustering-tree assessment across RNA-only, AbSeq-only, and WNN representations. Clustree diagrams show transitions in cell membership between clusters at adjacent resolution parameters for RNA-only (top; resolutions 0.3–0.9), AbSeq-only (middle; resolutions 0.3–0.9), and joint WNN (bottom; resolutions 0.3–1.0) representations. Nodes denote clusters at each resolution, node size reflects cell counts, and edges connect clusters that share cells across adjacent resolutions. RNA-only and AbSeq-only clustering used the Louvain algorithm (Seurat algorithm = 1), with selected resolutions of 0.5 and 0.4, respectively. WNN clustering used the smart local moving algorithm (SLM; Seurat algorithm = 3), with a selected resolution of 0.6. The selected settings yielded 25 RNA-only clusters, 17 AbSeq-only clusters, and 48 WNN graph clusters before artifact exclusion. Resolution selection also considered cluster size and RNA/AbSeq marker interpretability; clustree was used for visualization and assessment rather than as an automatic optimizer.

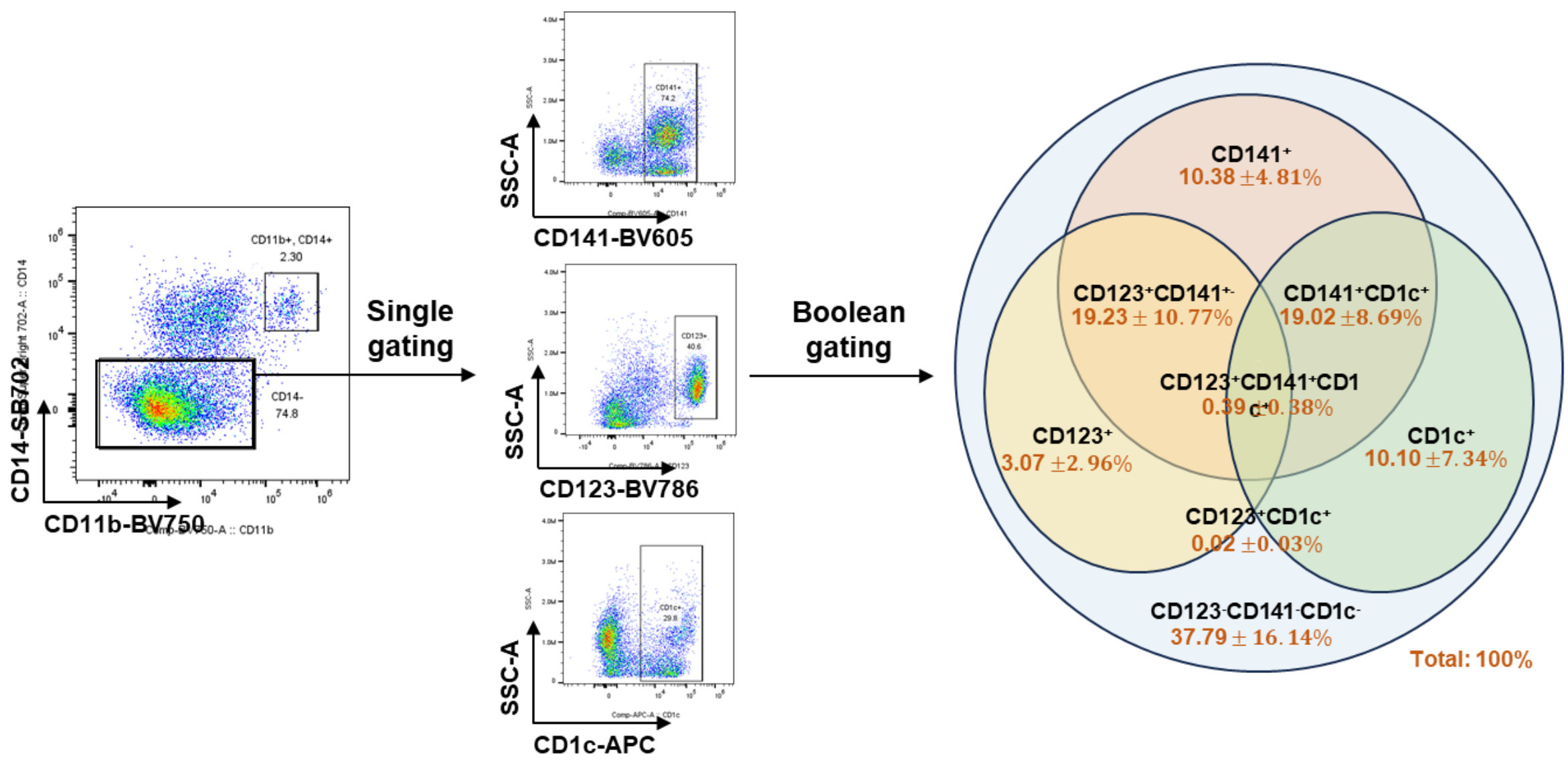

Supplementary Figure S3. Boolean gating of Lin$^-$HLA-DR$^+$CD14$^-$CD11b$^-$ cells. Lin$^-$CD14$^-$CD11b$^-$ cells gated from Lin-HLA$^-$DR$^+$ cells were single-gated into CD141$^+$, CD1c$^+$, and CD123$^+$ cells and Boolean-gated to detect combinations of surface marker expression. Data are mean ± SD (n = 5).

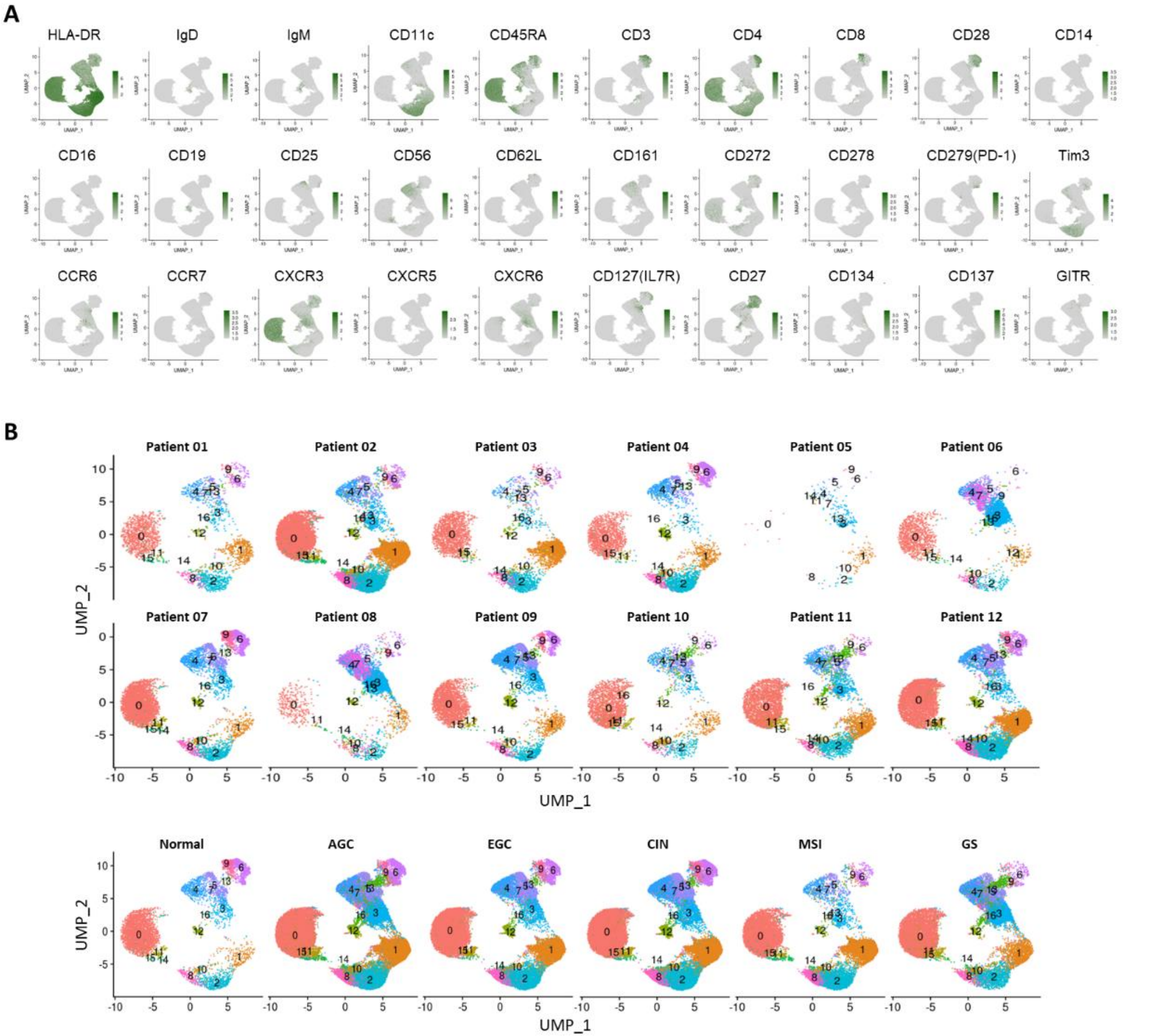

Supplementary Figure S4. Representative UMAP plots for the expression of 30 surface markers in ADT analysis. (A) UMAP of surface marker expression. (B) UMAP of 16 clusters in stages and molecular subtypes of 12 GC patients.

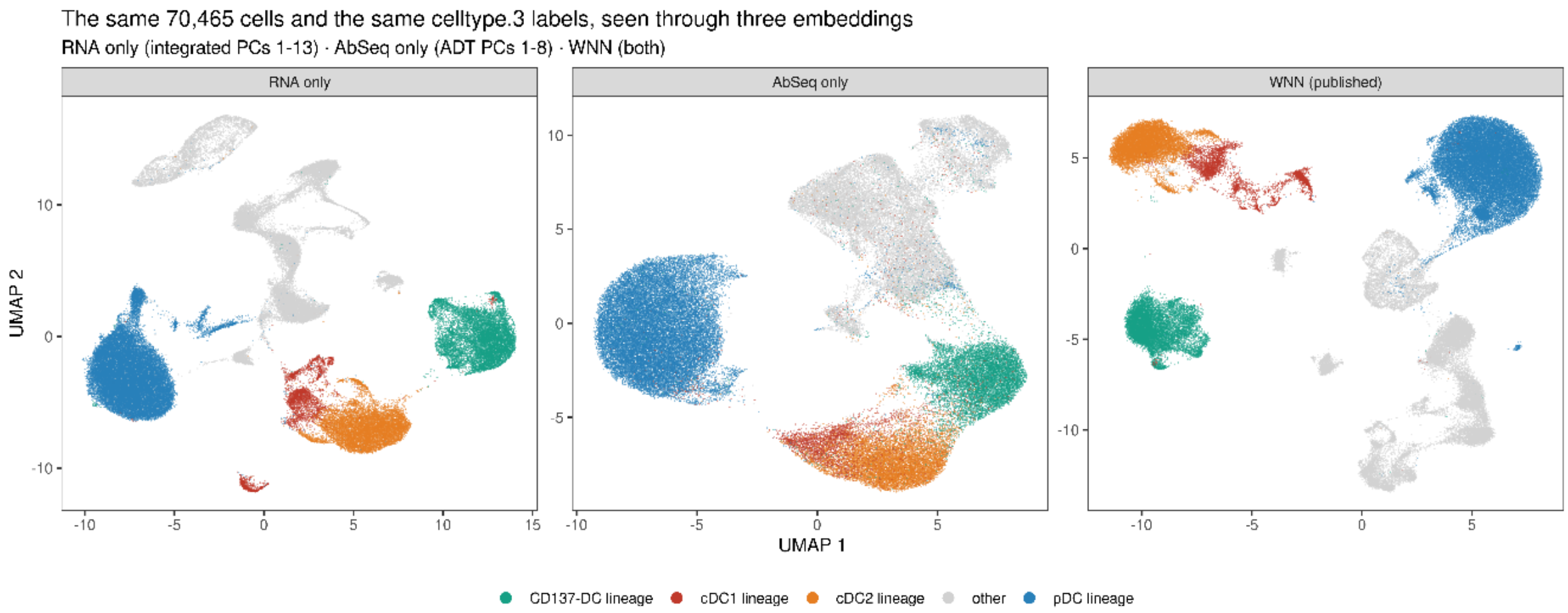

Supplementary Figure S5. Comparison of RNA-only, AbSeq-only, and joint WNN representations. UMAPs display the same 70,465 cells with the same archived lineage labels in the rPCA-integrated RNA-only representation (left; RNA principal components 1–13), the AbSeq-only representation (middle; ADT principal components 1–8), and the joint weighted nearest-neighbor representation (right; WNN). Cells are colored as CD137-DC lineage, cDC1 lineage, cDC2 lineage, pDC lineage, or other. The panels illustrate how the same annotated populations are arranged in each modality-specific or multimodal embedding; they are not independent classifier-performance plots.

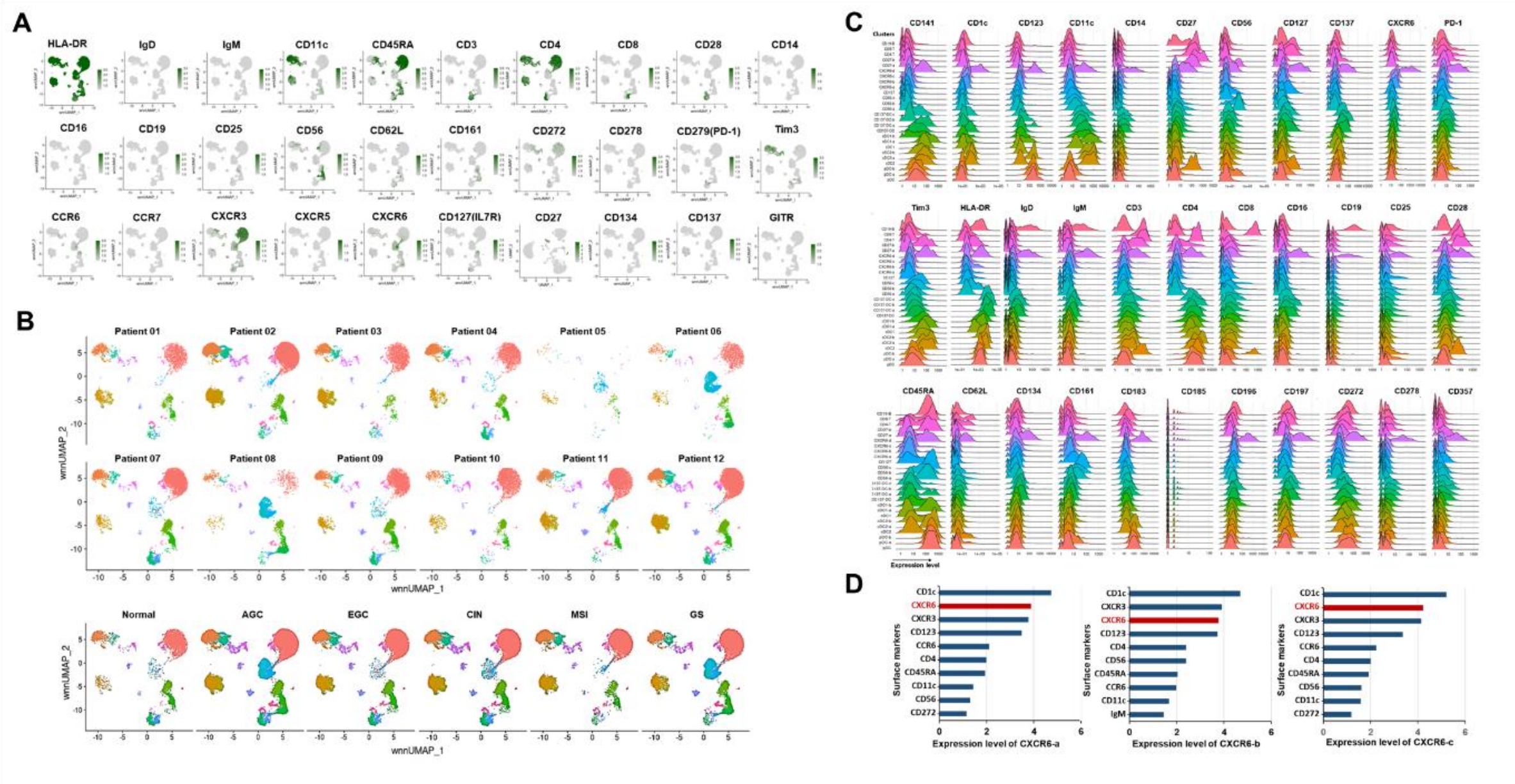

Supplementary Figure S6. Surface marker expression in 26 clusters identified by scRNA-seq with AbSeq. (A) UMAP showing expression of surface markers in clusters of Lin$^-$HLD-DR$^+$ cells by combined analysis of scRNA-seq with AbSeq. (B) UMAP of 26 clusters in clinical stages and molecular subtypes. (C) Ridge plots of surface marker expression across 26 clusters. (D) Comparative expression levels of surface markers in clusters of CXCR6 lineage.

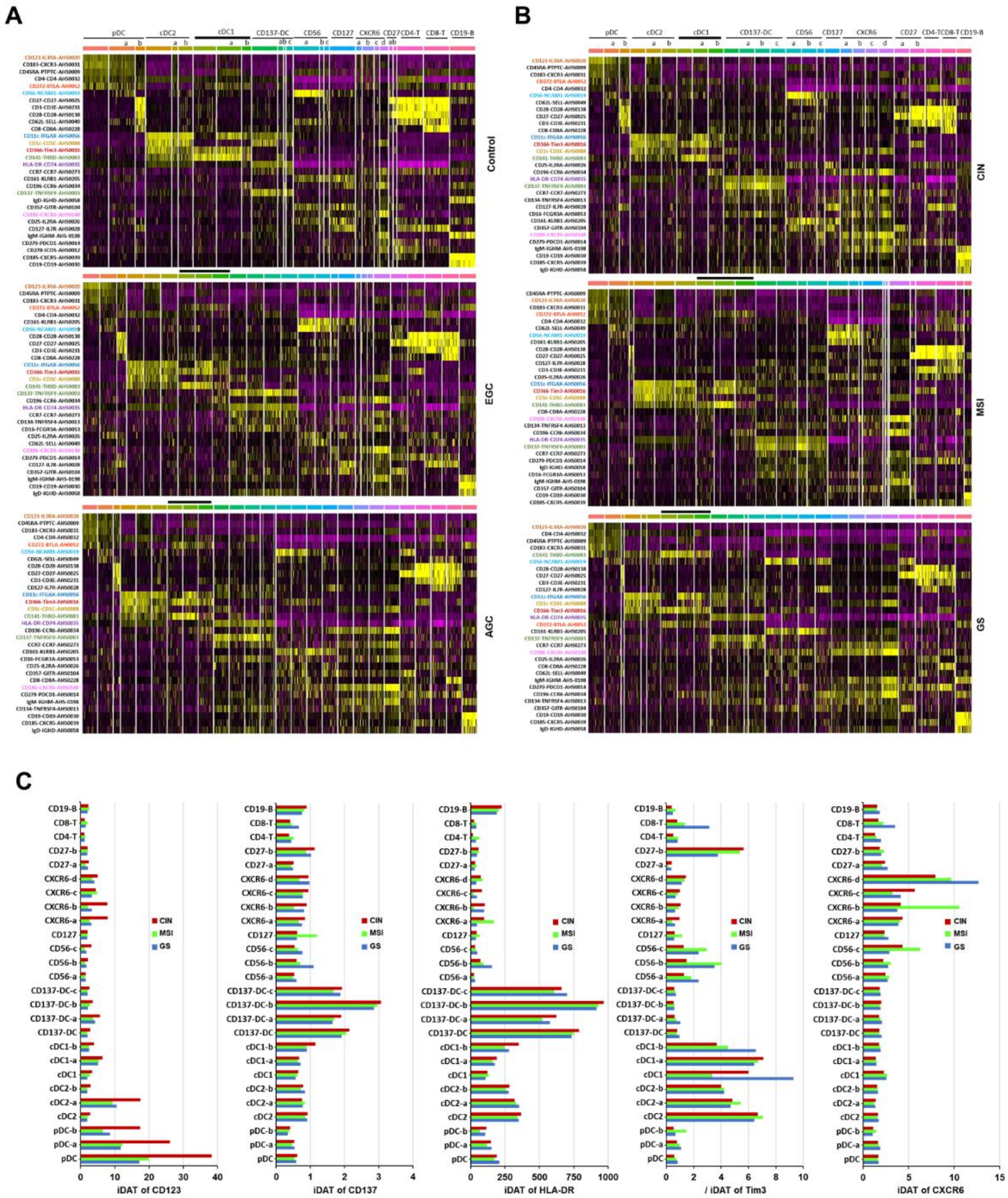

Supplementary Figure S7. Expression of major surface markers in clinical stages and molecular subtypes. (A, B) Heatmap showing surface marker expression by scRNA-seq and AbSeq across 26 clusters, by stage and molecular subtype. (C) Expression levels of surface markers in the 26 clusters of different molecular subtypes.

AGC, advanced gastric cancer; CIN, chromosomal instability; EGC, early gastric cancer; GS, genomically stable; MSI, microsatellite instability

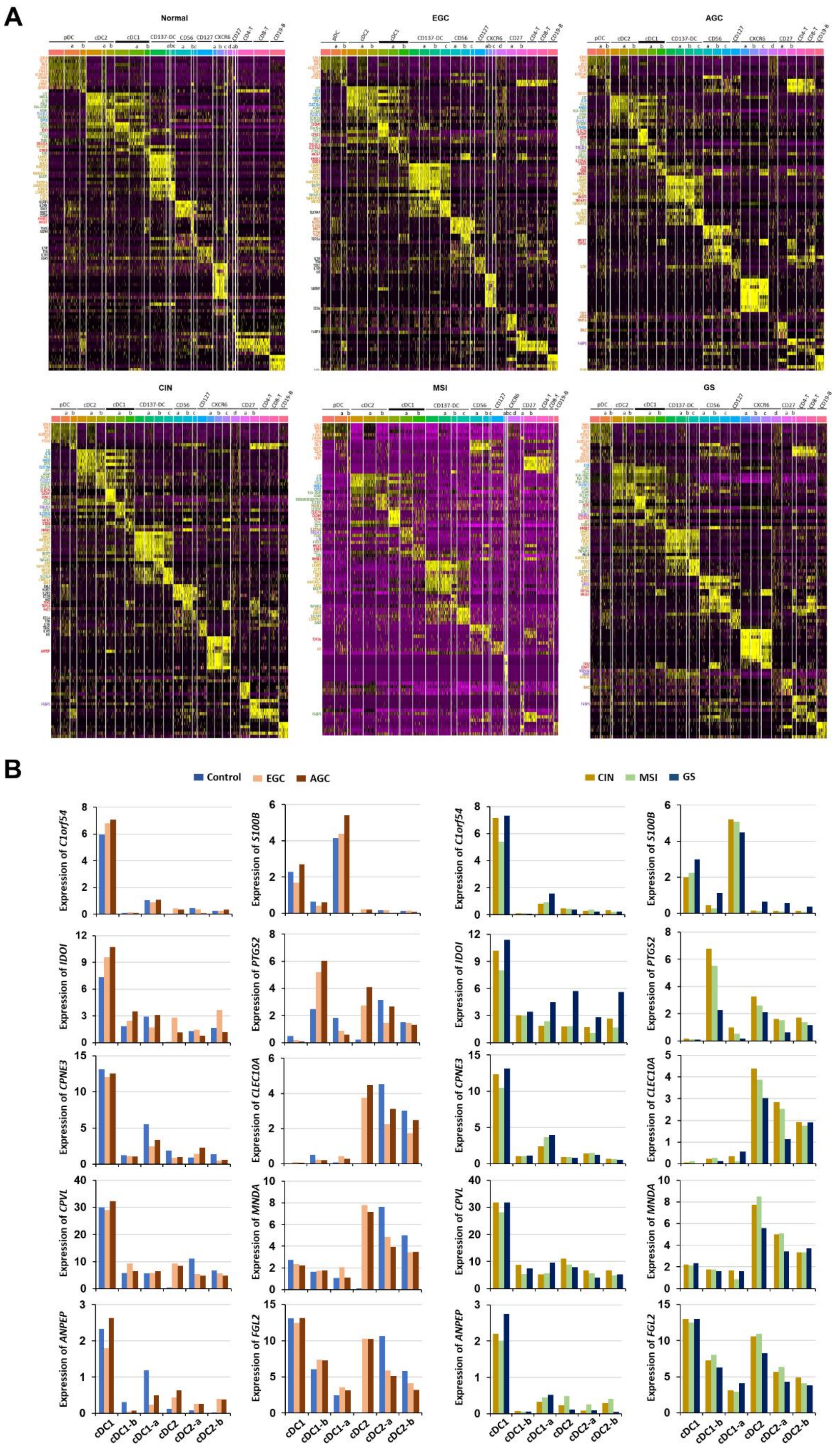
A
Normal
EGC
AGC
CIN
MSI
GS
pDC
cDC2
cDC1
CD137-DC
CD56
CD127
CXCR6
CD27
CD4-T
CD8-T
CD19-B
B
Control
EGC
AGC
CIN
MSI
GS
Expression of C1orf54
Expression of S100B
Expression of IDOI
Expression of PTGS2
Expression of CPNE3
Expression of CLEC10A
Expression of CPVL
Expression of MNDA
Expression of ANPEP
Expression of FGL2
cDC1
cDC1-b
cDC1-a
cDC2
cDC2-a
cDC2-b

Supplementary Figure S8. Gene expression within the same cluster by clinical stage and molecular pattern. (A) Heatmap of gene sets from 26 clusters, and (B) expression levels in the six clusters of the cDC1 and cDC2 lineages.

AGC, advanced gastric cancer; CIN, chromosomal instability; EGC, early gastric cancer; GS, genomically stable; MSI, microsatellite instability

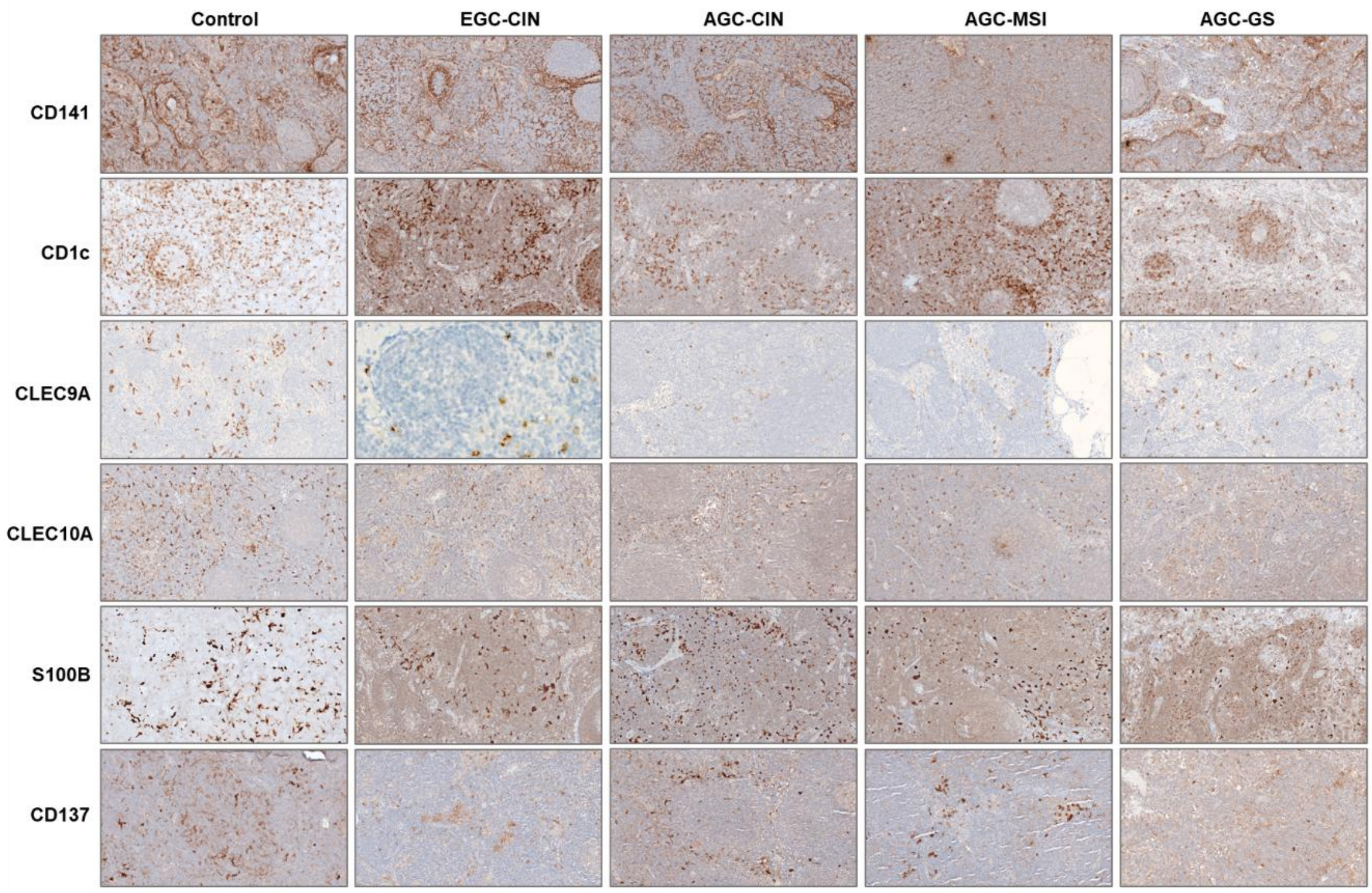

Supplementary Figure S9. Immunohistochemical findings of surface marker expression in LN (x400)**.**

AGC, advanced gastric cancer; CIN, chromosomal instability; EGC, early gastric cancer; GS, genomically stable; LN, lymph node; MSI, microsatellite instability.

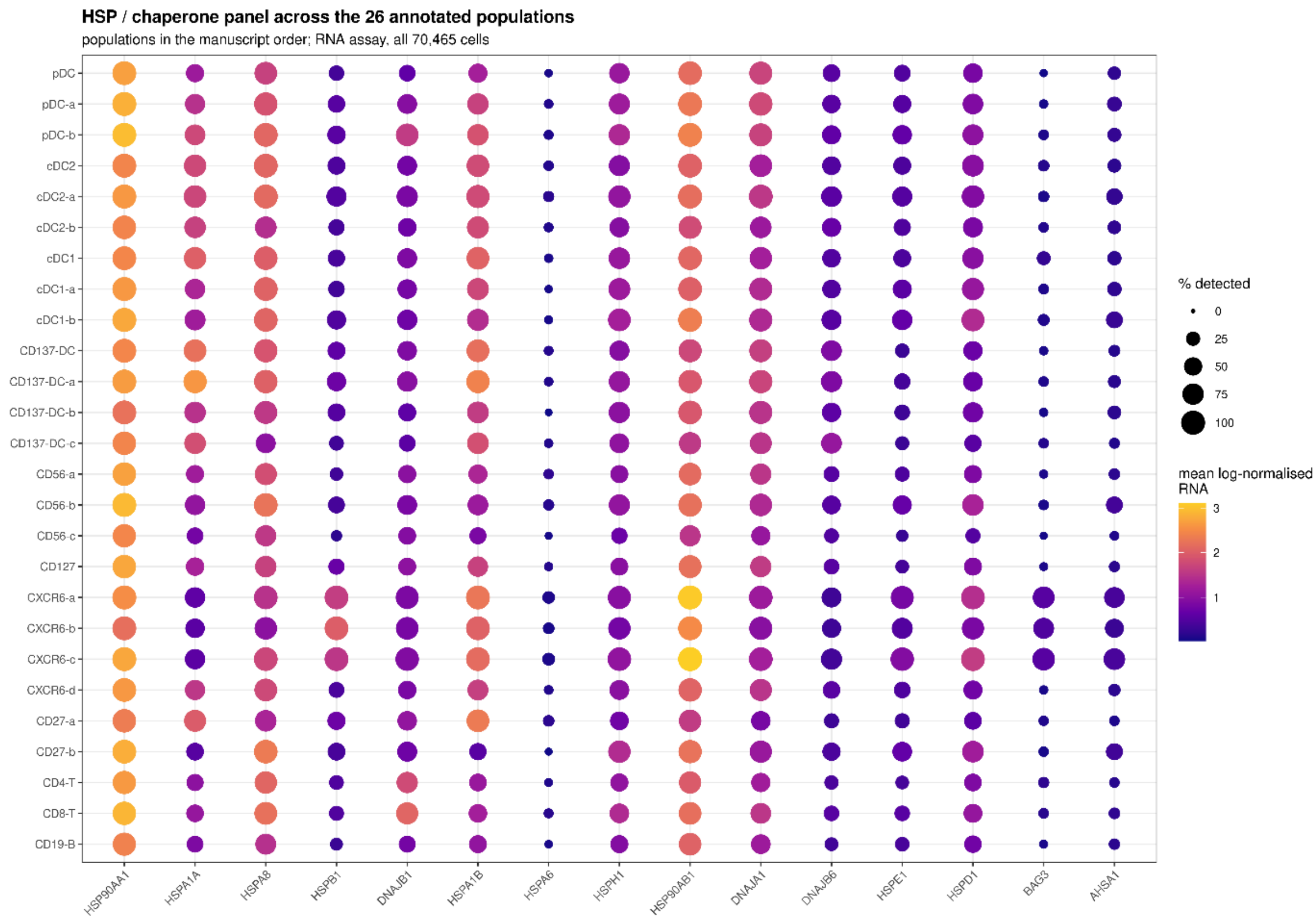

Supplementary Figure S10. Distribution of heat-shock and chaperone gene expression across the annotated cell populations. Dot plot showing RNA expression of 15 heat-shock/chaperone genes across the 26 archived populations in all 70,465 cells. Rows represent annotated populations, and columns represent *HSP90AA1, HSPA1A, HSPA8, HSPB1, DNAJB1, HSPA1B, HSPA6, HSPH1, HSP90AB1, DNAJA1, DNAJB6, HSPE1, HSPD1, BAG3,* and *AHSA1*. Dot size indicates the percentage of cells with detectable expression, and color indicates mean log-normalized RNA expression.

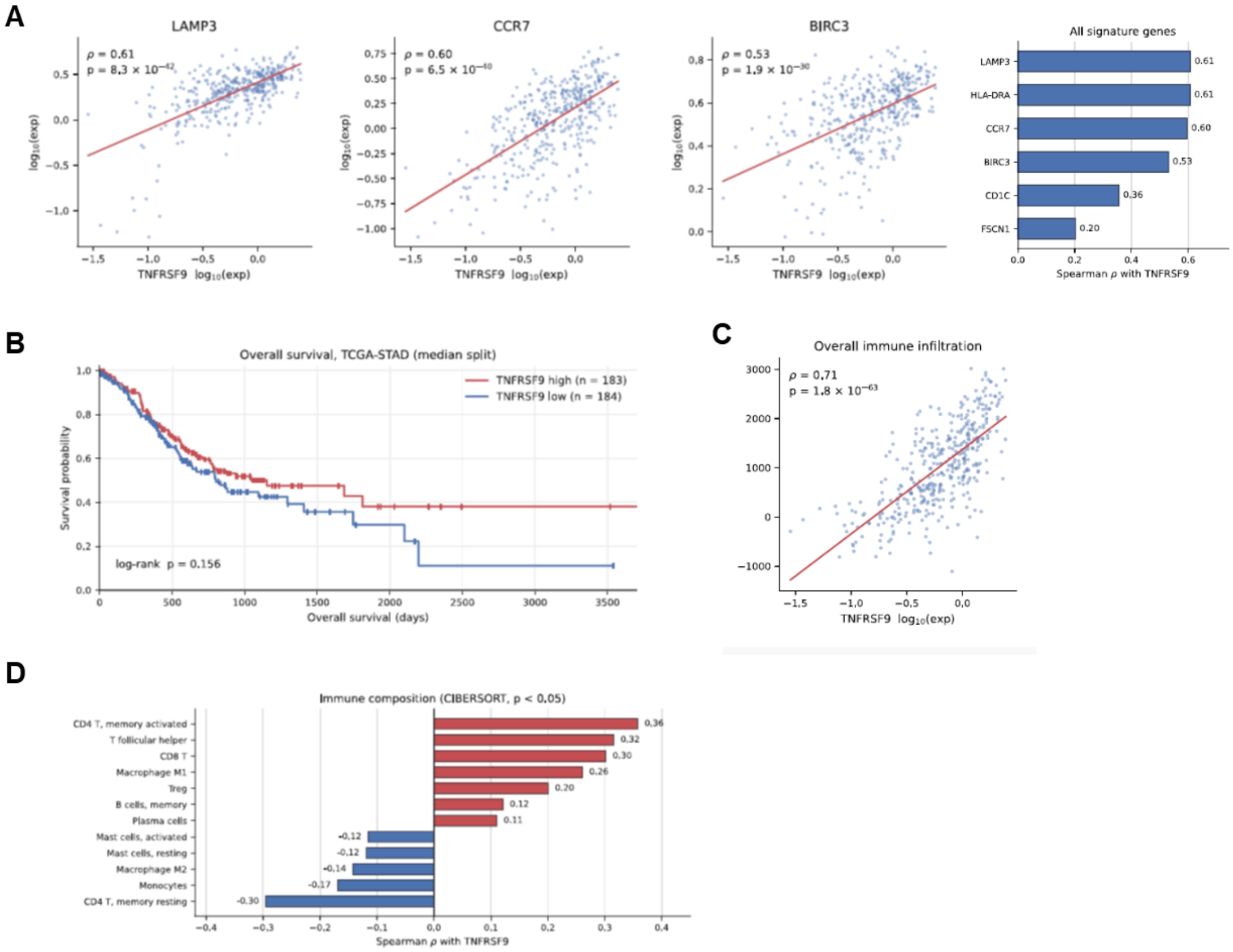

Supplementary Figure S11. ***TNFRSF9*** **expression in bulk gastric cancer tissue in TCGA-STAD.** (A) Correlation of *TNFRSF9* with genes of the CD137-DC signature. Scatter plots show *LAMP3*, *CCR7,* and *BIRC3* against *TNFRSF9* (log_{10}-transformed normalized expression); the line is a least-squares fit, and rho is the Spearman coefficient. The bar chart summarizes all six signature genes. (B) Overall survival by *TNFRSF9*, patients split at the median. p-value by log-rank test. (C) Correlation of *TNFRSF9* with the ESTIMATE ImmuneScore. (D) Spearman correlation between *TNFRSF9* and CIBERSORT-estimated immune cell fractions. Only cell types reaching $p < 0.05$ are shown; bars to the right of zero indicate a higher fraction in tumors with high *TNFRSF9*. Statistical analyses were performed in Python: Spearman rank correlation and two-sided log-rank survival tests.

Supplementary Table S1. **Clinicopathologic characteristics of patients with gastric cancer.**

Characteristic	No. (n=12)
Age at diagnosis, years	
Median (range)	70.5 (47–89)
Sex, n (%)	
Male	6 (50.0%)
Female	6 (50.0%)
Tumor location, n (%)	
Upper third	2 (16.7%)
Middle third	4 (33.3%)
Lower third	6 (50.0%)
Histologic type, n (%)	
Tubular adenocarcinoma	7 (58.3%)
Signet ring cell carcinoma	2 (16.7%)
Others	3 (25.0%)
Lauren classification, n (%)	
Intestinal	7 (58.3%)
Diffuse	2 (16.7%)
Mixed/ Indeterminate	3 (25.0%)
Tumor depth, n (%)	
EGC (pT1)	4 (33.3%)
AGC (≥pT2)	8 (66.7%)
Nodal status, n (%)	
N0	7 (58.3%)
N+ (pN1–3)	5 (41.7%)
Lymphatic invasion, n (%)	
Present	7 (58.3%)
Absent	5 (41.7%)
Venous invasion, n (%)	
Present	7 (58.3%)
Absent	5 (41.7%)
Perineural invasion, n (%)	
Present	8 (66.7%)
Absent	4 (33.3%)
Molecular subtype (TCGA classification), n (%)	

Chromosomal instability (CIN)	6 (50.0%)
Genomically stable (GS)	3 (25.0%)
Microsatellite instability (MSI)	3 (25.0%)
Epstein–Barr virus–positive (EBV)	0 (0%)
Helicobacter pylori infestation, n (%)	
Mild	8 (66.7%)
Absent	4 (33.3%)
Lymph node sampling characteristics	
Number of lymph node specimens per patient, median (range)	3.5 (1–4)
Lymph node weight (g), median (range)	0.245 (0.077–0.620)
Maximum lymph node size (mm), median (range)	12 (6–19)

Supplementary Table S2. Assignment of LN samples to sequencing batches and Sample Tags.

Batch	Patient ID	LN localization	Clinical information	TCGA grade	Sample Tag No.	Cells analyzed
**DC-set01**	1006_#1	Lesser curvature	AGC	CIN	No.1	2,748
**DC-set01**	1024_#8	Lesser curvature	AGC	MSI	No.2	14,647
**DC-set01**	1031_#12	Lesser curvature	EGC	CIN	No.3	4,298
**DC-set02**	1006_#1	Greater curvature	AGC	CIN	No.4	4,620
**DC-set02**	1006_#1	8a	AGC	CIN	No.5	380
**DC-set02**	1013_#4	Greater curvature	AGC	GS	No.6	4,282
**DC-set03**	0113_#3	-	Normal	Normal	No.7	7,314
**DC-set03**	1013_#4	9	AGC	GS	No.8	4,658
**DC-set03**	1011_#2	8a	EGC	CIN	No.9	6,548
**DC-set04**	1017_#6	Lesser curvature	AGC	GS	No.10	3,356
**DC-set04**	1017_#6	8a	AGC	GS	No.11	6,205
**DC-set04**	1031_#12	8a	EGC	CIN	No.12	11,409

AGC, advanced gastric cancer; CIN, chromosomal instability; EGC, early gastric cancer; GS, genomically stable; LN, lymph node; MSI, microsatellite instability.

Supplementary Table S3. Abs used in flow cytometry.

Product Name	Fluorescence	Catalog No	Company
LIVE/DEAD AQUA		L34957	Thermo Fisher Scientific
CD45	FITC	11-0459-42	Thermo Fisher Scientific
CD3	PE-Cy5.5	35-0036-42	Thermo Fisher Scientific
CD19	PE-eFluor 610	61-0198-42	Thermo Fisher Scientific
CD14	Super Bright 702	67-0149-42	Thermo Fisher Scientific
HLA-DR	APC-eFluor 780	47-9956-42	Thermo Fisher Scientific
CD11c	Alexa Fluor 700	56-0116-42	Thermo Fisher Scientific
CD11b	BV750	747357	BD Biosciences
CD141	BV605	740421	BD Biosciences
XCR1	BV421	372610	Biolegend
CD1c	APC	331524	Biolegend
CD1a	PE	12-0017-42	Thermo Fisher Scientific
CD123	BV786	564196	BD Biosciences
CD83	PE-Cy7	25-0839-42	Thermo Fisher Scientific
PD-L1	PE-Fire 810	329755	Biolegend
PD-1	PE-Cy5	15-2799-42	Thermo Fisher Scientific

Supplementary Table S4. 33 pre-titrated Abs used in AbSeq.

No	Specificity	Clone	Oligo ID	No	Specificity	Clone	Oligo ID	No	Specificity	Clone	Oligo ID
**1**	CD123	7G3	AHS0020	**12**	CD27	M-T271	AHS0025	**23**	CD186 (CXCR6)	13B 1E5	AHS014
**2**	CD1c	F10/21A3	AHS0088	**13**	CD28	L293	AHS0138	**24**	CD196 (CCR6)	11A9	AHS0034
**3**	CD141	1A4	AHS0083	**14**	CD45RA	HI100	AHS0009	**25**	CD197 (CCR7)	2-L1-A	AHS0273
**4**	CD3	UCHT1	AHS0231	**15**	CD56	NCAM16	AHS0019	**26**	CD272	J168-540	AHS0052
**5**	CD4	SK3	AHS0032	**16**	CD62L	DREG-56	AHS0049	**27**	CD278	DX29	AHS0012
**6**	CD8	SK1	AHS0228	**17**	CD127	HIL-7R-M21	AHS0028	**28**	CD279	EH12.1	AHS0014
**7**	CD11c	B-Ly6	AHS0056	**18**	CD134	ACT35	AHS0013	**29**	CD357	V27-580	AHS0104
**8**	CD14	MPHIP9	AHS0037	**19**	CD137	4B4-1	AHS0003	**30**	CD366	7D3	AHS0016
**9**	CD16	3G8	AHS0053	**20**	CD161	HP-3G10	AHS0205	**31**	HLA-DR	G46-6	AHS0035
**10**	CD19	SJ25C1	AHS0030	**21**	CD183 (CXCR3)	1C6/CXCR3	AHS0031	**32**	IgD	IA6-2	AHS0058
**11**	CD25	2A3	AHS0026	**22**	CD185	RF8B2	AHS0039	**33**	IgM	G20-127	AHS0198

Supplementary Table S5. Run-specific quality-control thresholds applied prior to data integration across the four scRNA-seq processing/sequencing runs.

Processing/sequencing run	nFeature_RNA lower cutoff	nFeature_RNA upper cutoff	percent.mt cutoff
**DC-set01**	>1,000	<7,500	<30%
**DC-set02**	>500	Not applied	<30%
**DC-set03**	>500	Not applied	<30%
**DC-set04**	>500	<6,000	<30%

Supplementary Table S6. Run-specific cell classification

Run	Called	Singlet	Multiplet	Undetermined
**DC-set01**	24,931	22,379 (89.76%)	1,440 (5.78%)	1,112 (4.46%)
**DC-set02**	11,048	9,774 (88.47%)	660 (5.97%)	614 (5.56%)
**DC-set03**	22,794	19,609 (86.03%)	1,056 (4.63%)	2,129 (9.34%)
**DC-set04**	23,787	21,501 (90.39%)	801 (3.37%)	1,485 (6.24%)
**Total**	82,560	73,263 (88.74%)	3,957 (4.79%)	5,340 (6.47%)

Supplementary Table S7. Cluster resolution and counts.

View	Resolutions evaluated	Selected	Clusters	Algorithm
RNA-only	0.3–0.9<br>(0.1 steps)	0.5	25	Louvain (1)
ADT-only	0.3–0.9<br>(0.1 steps)	0.4	17	Louvain (1)
WNN	0.3–1.0<br>(0.1 steps); extended to 0.05–1.0	0.6	48<br>before exclusion	SLM (3)

Supplementary Table S8. Population of pDC and cDC subtypes in HLA-DR^{+}CD14^{-}CD11b^{-} cells isolated from LNs of GC patients.

<table>
<tr><th>Subtype</th><th>%</th><th>Subtype</th><th>%</th><th>Subtype</th><th>%</th><th>Subtype</th><th>%</th><th>Subtype</th><th>Name</th></tr>
<tr><td rowspan="2">CD123^{+}
(G1)</td><td rowspan="2">14.7±7.8
(0.13±0.10)</td><td rowspan="2">CD141$^{+/-}$</td><td rowspan="2">100
(0.13±0.10)</td><td>CD1c^{+}</td><td>2.3±0.1
(0)</td><td>CD11c^{+}</td><td>0</td><td></td><td></td></tr>
<tr><td>CD1c^{-}
(F0)</td><td>97.6±0.3
(0.13±0.1)</td><td>CD11c^{-}</td><td>100
(0.13±0.10)</td><td>CD123^{+}CD141$^{+/-}$CD1c^{-}
CD11c^{-}</td><td>pDC</td></tr>
<tr><td rowspan="7">CD123^{-}
(G2+G3)</td><td rowspan="7">84.5±7.9
(0.66±0.29)</td><td rowspan="4">CD141^{+}
(G2)</td><td rowspan="4">42.6±11.4
(0.34±0.12)</td><td rowspan="2">CD1c^{+}
(F1)</td><td rowspan="2">53.3±11.0
(0.18±0.09)</td><td>CD11c^{+}</td><td>74.2±14.5
(0.14±0.09)</td><td>CD123^{-}
CD141^{+}
CD1c^{+}CD11c^{+}</td><td>DP-p</td></tr>
<tr><td>CD11c^{-}</td><td>25.8±14.4
(0.05±0.04)</td><td>CD123^{-}
CD141^{+}
CD1c^{+}CD11c^{-}</td><td>DP-n</td></tr>
<tr><td rowspan="2">CD1c^{-}
(F2)</td><td rowspan="2">46.7±11.0
(0.15±0.08)</td><td>CD11c^{+}</td><td>42.1±16.9
(0.07±0.05)</td><td>CD123^{-}D141^{+}
CD1c^{-}CD11c^{+}</td><td>cDC1-p</td></tr>
<tr><td>CD11c^{-}</td><td>57.9±16.9
(0.09±0.04)</td><td>CD123^{-}
CD141^{+}
CD1c^{-}CD11c^{-}</td><td>cDC1</td></tr>
<tr><td rowspan="3">CD141^{-}
(G3)</td><td rowspan="3">41.9±14.4
(0.32±0.24)</td><td rowspan="2">CD1c^{+}
(F3)</td><td rowspan="2">22.4±11.6
(0.08±0.05)</td><td>CD11c^{+}</td><td>16.2±10.1
(0.01±0.01)</td><td>CD123^{-}D141^{-}
CD1c^{+}CD11c^{+}</td><td>cDC2-p</td></tr>
<tr><td>CD11c^{-}</td><td>83.8±10.5
(0.07±0.05)</td><td>CD123^{-}D141^{-}
CD1c^{+} CD11c^{-}</td><td>cDC2</td></tr>
<tr><td>CD1c^{-}
(F4)</td><td>77.8±11.6
(0.25±0.23)</td><td>CD11c^{+}</td><td>17.9±12.3
(0.06±0.07)</td><td>CD123^{-}D141^{-}
CD1c^{-}CD11c^{+}</td><td>DN-p</td></tr>
</table>

						CD11c^{-}	82.1±12.3 (0.19±0.18)	CD123^{-}D141^{-} CD1c^{-}CD11c^{-}	DN-n
Total (%)*	(0.79±0.35)		(0.79)		(0.79)		(0.79)		

* DP, CD141^{+}CD1c^{+} double-positive cell; DN, CD141^{-}CD1c^{-} double negative cell; p, CD11c^{+} cell; n, CD11c^{-} cell.
**Numbers in parentheses are positive cells (%) among total LN cells
Data represent Mean ± SD (n = 33).

Supplementary Table S9. Population of pDC and cDC subsets in HLA-DR^{+}CD14^{-}CD11b^{-} cells isolated from the LN of controls.

Subtype	%	Subtype	%	Subtype	%	Subtype	%	Subtype	Name
CD123^{+} (G1)	16.8±8.1 (0.45±0.10)	CD141$^{+/-}$	100 (0.13±0.10)	CD1c^{+}	0.3±0.1 (0)	CD11c^{+}	0		
				CD1c^{-} (F0)	99.7±0.3 (0.13±0.1)	CD11c^{-}	100 (0.13±0.10)	CD123^{+}CD141$^{+/-}$ CD1c^{-}CD11c^{-}	pDC
CD123^{-} (G2+G3)	82.3±8.1 (2.2±0.29)	CD141^{+} (G2)	34.6±11.2 (0.93±0.12)	CD1c^{+} (F1)	57.9±11.6 (0.54±0.09)	CD11c^{+}	82.3±14.0 (0.45±0.07)	CD123^{-}CD141^{+} CD1c^{+}CD11c^{+}	DP-p
						CD11c^{-}	17.7±14.0 (0.10±0.03)	CD123^{-}CD141^{+} CD1c^{+}CD11c^{-}	DP-n
				CD1c^{-} (F2)	42.1±11.7 (0.39±0.08)	CD11c^{+}	65.7±17.0 (0.07±0.05)	CD123^{-}D141^{+} CD1c^{-}CD11c^{+}	cDC1-p
						CD11c^{-}	34.2±17.0 (0.13±0.06)	CD123^{-}CD141^{+} CD1c^{-}CD11c^{-}	cDC1
		CD141^{-} (G3)	47.7±14.4 (1.29±0.24)	CD1c^{+} (F3)	33.4±13.1 (0.34±0.05)	CD11c^{+}	11.5±10.5 (0.05±0.01)	CD123^{-}D141^{-} CD1c^{+}CD11c^{+}	cDC2-p
						CD11c^{-}	88.5±10.5 (0.38±0.05)	CD123^{-}D141^{-} CD1c^{+} CD11c^{-}	cDC2
				CD1c^{-} (F4)	77.8±11.6 (0.86±0.23)	CD11c^{+}	21.7±13.6 (0.19±0.07)	CD123^{-}D141^{-} CD1c^{-}CD11c^{+}	DN-p
						CD11c^{-}	78.3±13.6 (0.67±0.18)	CD123^{-}D141^{-} CD1c^{-}CD11c^{-}	DN-n
Total (%)*	(2.65±0.25)		(2.65)		(2.65)		(2.65)		

* Positive cells (%) among total LN cells
Data represent Mean ± SD (n = 3).
